# Latency Entry of Herpes Simplex Virus 1 Is Determined by the Interaction of Its Genome with the Nuclear Environment

**DOI:** 10.1371/journal.ppat.1005834

**Published:** 2016-09-12

**Authors:** Mohamed Ali Maroui, Aleth Callé, Camille Cohen, Nathalie Streichenberger, Pascale Texier, Julie Takissian, Antoine Rousseau, Nolwenn Poccardi, Jérémy Welsch, Armelle Corpet, Laurent Schaeffer, Marc Labetoulle, Patrick Lomonte

**Affiliations:** 1 Univ Lyon, Université Claude Bernard Lyon 1, CNRS UMR 5310, INSERM U 1217, LabEx DEVweCAN, Institut NeuroMyoGène (INMG), team Chromatin Assembly, Nuclear Domains, Virus, Lyon, France; 2 Univ Lyon, Université Claude Bernard Lyon 1, CNRS UMR 5310, INSERM U 1217, Institut NeuroMyoGène (INMG), team Nerve-Muscle Interactions, Lyon, France; 3 Univ Lyon, Centre Hospitalier Universitaire de Lyon, Hospices Civils de Lyon, Centre de Pathologie et Neuropathologie Est, Bron, France; 4 Institut de Biologie Intégrative de la Cellule (I2BC), Département de Virologie, Gif-sur-Yvette, France; 5 Université Paris Sud, Centre Hospitalier Universitaire de Bicêtre, Service d'Ophthalmologie, Le Kremlin-Bicêtre, France; 6 Ecole Normale Supérieure de Lyon, CNRS UMR 5308, INSERM U 1111, Centre International de Recherche en Infectiologie (CIRI), team Immunobiologie des infections virales, Lyon, France; La Jolla Institute for Allergy and Immunology, UNITED STATES

## Abstract

Herpes simplex virus 1 (HSV-1) establishes latency in trigeminal ganglia (TG) sensory neurons of infected individuals. The commitment of infected neurons toward the viral lytic or latent transcriptional program is likely to depend on both viral and cellular factors, and to differ among individual neurons. In this study, we used a mouse model of HSV-1 infection to investigate the relationship between viral genomes and the nuclear environment in terms of the establishment of latency. During acute infection, viral genomes show two major patterns: replication compartments or multiple spots distributed in the nucleoplasm (namely “multiple-acute”). Viral genomes in the “multiple-acute” pattern are systematically associated with the promyelocytic leukemia (PML) protein in structures designated viral DNA-containing PML nuclear bodies (vDCP-NBs). To investigate the viral and cellular features that favor the acquisition of the latency-associated viral genome patterns, we infected mouse primary TG neurons from wild type (wt) mice or knock-out mice for type 1 interferon (IFN) receptor with wt or a mutant HSV-1, which is unable to replicate due to the synthesis of a non-functional ICP4, the major virus transactivator. We found that the inability of the virus to initiate the lytic program combined to its inability to synthesize a functional ICP0, are the two viral features leading to the formation of vDCP-NBs. The formation of the “multiple-latency” pattern is favored by the type 1 IFN signaling pathway in the context of neurons infected by a virus able to replicate through the expression of a functional ICP4 but unable to express functional VP16 and ICP0. Analyses of TGs harvested from HSV-1 latently infected humans showed that viral genomes and PML occupy similar nuclear areas in infected neurons, eventually forming vDCP-NB-like structures. Overall our study designates PML protein and PML-NBs to be major cellular components involved in the control of HSV-1 latency, probably during the entire life of an individual.

## Author Summary

Establishment of latency of herpes simplex virus 1 (HSV-1) at the cellular level results from the combination of a series of complex molecular events involving cellular and viral-associated features. HSV-1 establishes latency in trigeminal ganglia (TG) sensory neurons. HSV-1 genomes remain as extrachromosomal DNA; their initial interaction with the nuclear architecture is likely to determine commitment toward the lytic or the latent transcriptional program. Among the major nuclear components that influence the infection process the promyelocytic leukemia (PML) nuclear bodies (NBs) play a major role as nuclear relays of the intrinsic antiviral response. In this study, using infected mice and cultured mouse primary TG neuron models, as well as human TGs, we investigated the interaction between HSV-1 genomes and the nuclear environment in individual neurons. We found that the inability of HSV-1 to initiate a lytic program at the initial stages of infection led to the formation of latency-associated viral DNA-containing PML-NBs (vDCP-NBs), or another pattern if the type 1 interferon pathway was activated prior to infection. vDCP-NB–like structures were also present in neurons of latently infected human TGs, designating PML-NBs as major nuclear components involved in the control of HSV-1 latency for the entire life of an individual.

## Introduction

Herpes simplex virus 1 (HSV-1) is a neurotropic virus that establishes a life-long latent infection in the trigeminal ganglia (TG) (or Gasserian ganglia) of the infected human host. From time to time the virus asymptomatically or symptomatically reactivates from the latency stage producing epithelial lesions, most of the time on the face but also in the eye, inducing severe pathologies such as keratitis [1]. HSV-1 infection is also associated with pathologies of the central nervous system (CNS), such as encephalitis, especially after primary infection of newborn children with deficiencies in their innate immunity due to genetic alteration of two genes coding proteins involved in the intrinsic antiviral response [2]. In mouse models reproducing latent infection, HSV-1 has also been shown to lead to brain pathologies following reactivation through retrograde transport of the viral particles towards the CNS [3,4].

During latency the virus is in a transcriptionally restricted state. Of the about 80 genes transcribed during lytic infection, only a family of long non-coding RNAs is produced abundantly during latency. These latency associated transcripts (LATs) arise from the transcription of an 8.3 kb primary RNA that is processed in two major LATs of 1.5 kb and 2 kb and several microRNAs with cellular and viral targets [5–12]. The precise role of LATs is a matter of debate; however, a point of convergence among the many studies of LATs is that their initial production would favor the survival of the infected neurons and the coordination of the infectious process towards the latency transcriptional program and reactivation [13–17]. The lytic cycle is the alternative transcriptional program and is characterized by a temporarily regulated transcriptional program, which starts with the expression of immediate early (IE), then early (E), and finally late (L) genes (reviewed in [18,19]). Three proteins favor the onset of the lytic cycle, namely ICP4, ICP0, and VP16. ICP4 is an IE protein and the major viral transactivator that induces the transcription of viral genes of all kinetics [20]. ICP4 is essential for the virus to enter the replication stage and for productive infection [21]. ICP0 is also an IE protein. ICP0 is a RING-finger protein that possesses SUMO-targeted E3 ubiquitin ligase (STUbL) activity [22,23]. ICP0 induces the proteasomal degradation of many cellular proteins, including components of the promyelocytic leukemia nuclear bodies (PML-NBs or ND10), and centromeres [24–29]. As a consequence, ICP0 induces the destabilization of PML-NBs and centromere chromatin, which contributes to creation of a nuclear environment suitable for lytic infection [29–34]. VP16 (α-TIF) is a virion-associated multifunctional protein that transactivates the expression of the five viral IE genes through its interaction with two cellular proteins, HCF-1, a cell cycle regulator and Oct-1, a transcription factor [35–40].

In the viral particle, HSV-1 genome is a 150-kb double stranded naked linear DNA. Upon entry into the nucleus, the viral genome does not integrate in the host cell genome, instead remaining as an extrachromosomal entity. As such, it sustains a process of circularization and associates with chromatin remodeling factors to be chromatinized [41–43]. Chromatinization of the viral genome during latency plays a major regulatory role, and post-translational modifications of histones associated to key viral promoters determines the fate of the latency/reactivation process [39,42,43]. However, latent viral genomes are present in multiple copies within the nucleus of infected neurons in mouse models and human [44–46], and little is known about the molecular determinants that enable one neuron rather than another to sustain reactivation. In contrast, within an individually reactivating neuron, whether some viral genomes are more prone to lead to a complete lytic transcriptional program is unknown. The question is legitimate since in a recent study we reported that the viral genomes were non-randomly distributed in the nucleus of latently infected mouse TG neurons [47]. Latent viral genomes showed two major patterns namely “single” (a single viral genome spot detected in the nucleus) and “multiple-latency” (up to 20–30 spots detected) differentially distributed in the nucleus. The “single” pattern was exclusively associated with the promyelocytic leukemia nuclear bodies (PML-NBs), forming structures known as viral DNA-containing PML-NBs or vDCP-NBs. In the “multiple-latency” pattern some viral genomes co-localized with PML-NBs, while others co-localized with centromeres, or were distributed in the nucleoplasm distal from PML-NBs and centromeres. Importantly, the expression of LATs from individual genomes was observed only for viral genomes neither associated with PML-NBs nor with centromeres. These data highlighted the previously anticipated heterogeneity of HSV-1 latency at the molecular level, and confirmed the major role played by the nuclear environment in the maintenance of latency and probably the reactivation process.

In the present study, using a fluorescent *in situ* hybridization (FISH) approach combined to immunofluorescence (IF), we investigated the interaction between viral genomes and nuclear proteins within TG neurons of latently infected mice and during the whole process of latency establishment (from 4 to 28 days post infection, dpi). We detected viral genomes in neurons and satellite cells at 4 and 6 dpi, but only in neurons at > 6 dpi. In satellite cells, viral genomes showed only replication compartment (RC) patterns, whereas in neurons both RC and “multiple-acute” patterns were detected. From 4 to 14 dpi both patterns progressively disappeared, and transformed from14dpi onwards to the latency-associated “single” and “multiple-latency” patterns. Expression of two lytic program-associated proteins, ICP4 and ICP27, was detected only in cells with the RC pattern. LAT expression was detected in “multiple-latency” but not “multiple-acute” pattern-containing neurons. Interestingly, at 4 to 8 dpi, a subset of RC-containing neurons showed LAT expression. The “multiple-acute” viral genomes co-localized with PML, Daxx, ATRX, SUMO-1 and SUMO-2/3 proteins in structures similar to vDCP-NBs but with a difference in number per infected neurons (up to 10 vDCP-NBs/neuron at 6 dpi). To gain a better insight into the cellular and viral factors that could lead to the formation of vDCP-NBs or “multiple-latency” patterns, cultures of mouse primary TG neurons from wt mice or knock-out mice for the type I interferon (IFN) receptor were infected with wt or temperature-sensitive (ts) mutant viruses. The results indicates that defects in the onset of the lytic program due to the absence of functional ICP4, combined with the absence of functional ICP0 were the two viral features that led to the formation of vDCP-NBs. In contrast, the type I IFN signaling pathway was required for the formation of a “multiple-latency”-like pattern, demonstrating the essential role of innate immunity in the acquisition of latency-associated viral genome patterns. Finally, immuno-FISH analyses of human TG showed a close spatial distribution between latent HSV-1 genomes and PML protein in neurons, which suggests that, similar to the situation in the mouse model, HSV-1 latency in human is probably tightly linked to the activity of PML-NBs.

## Results

### Nuclear distribution of viral genomes during establishment of latency

**Fig 1:**
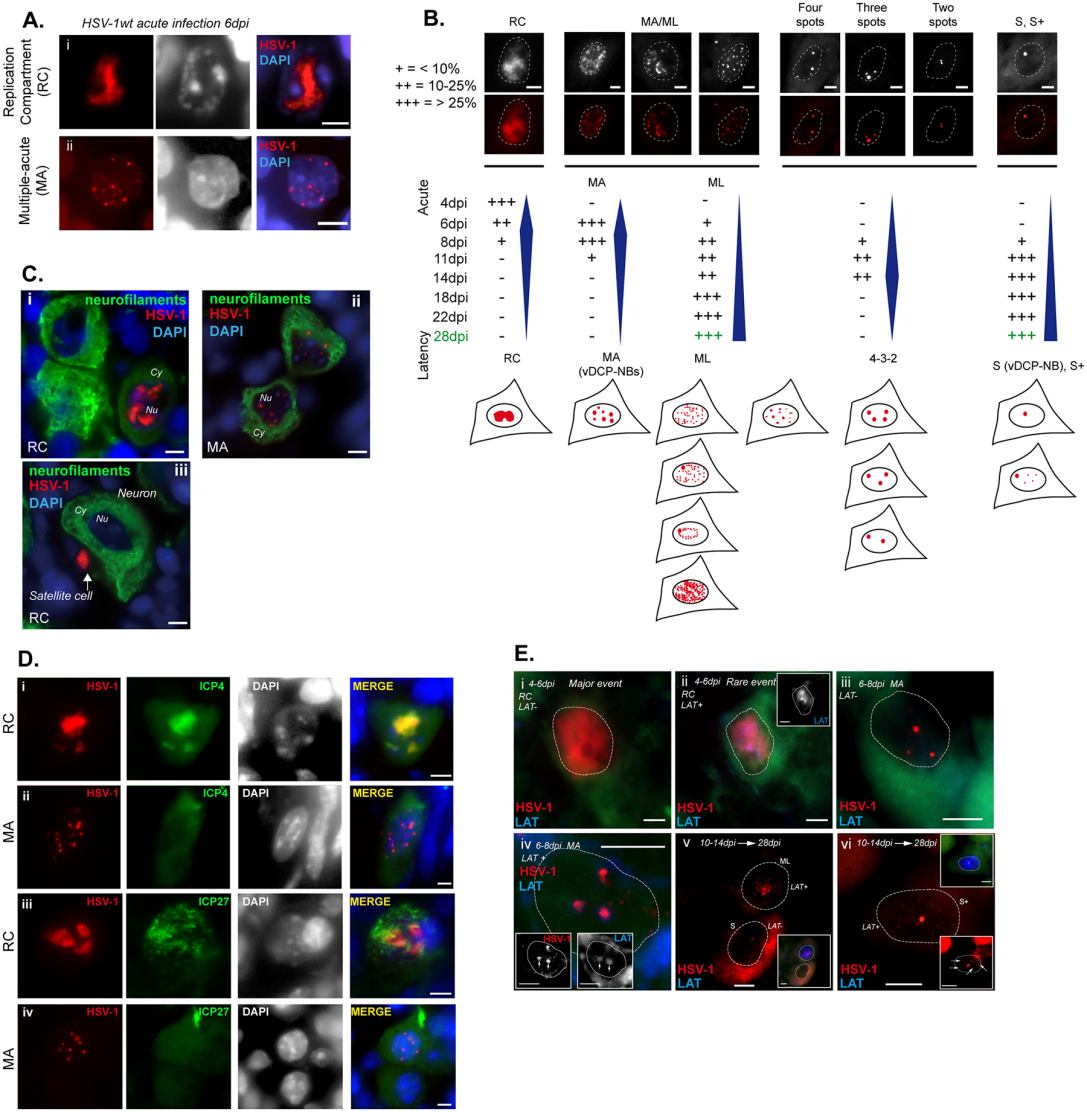
Characterization of herpes simplex virus 1 (HSV-1) genomes during establishment of latency. (A) DNA-FISH detection of HSV-1 genomes (red). (i) HSV-1 replication compartment (RC) pattern (ii) HSV-1 multiple-acute (MA) pattern. Black/white middle images represent staining of the cellular DNA with DAPI. (B) The HSV-1 genome patterns detected during establishment of latency (from 4 to 28 dpi) are presented as colored and black-and-white DNA-FISH images (up), and drawings (down). Patterns detected were: RC; MA; multiple-latency (ML); four, three, two spots (4-3-2); and single (S) or single+ (S+). The relative proportions of each pattern are signified by “+” or “–“.“-“: pattern was not observed; “+”: less than 10% of the observed patterns; “++”: between 10% and 25% of the patterns; and “+++”: more than 25% of the patterns. Dashed lines indicate nuclei. (C) Immuno-DNA-FISH showing HSV-1 genomes (red, 6 dpi), neurofilament (NF160) protein (green), and cellular chromatin (DAPI, blue). Nu: nucleus; Cy: cytoplasm. (D) Immuno-DNA-FISH showing HSV-1 genomes (red, 6 dpi), viral proteins (green), and cellular chromatin (DAPI, blue/grey). (E) RNA-DNA FISH showing HSV-1 genomes (red) and LAT (blue/grey). Inset at top right in (vi) shows LAT expression; inset at bottom right in (vi) shows the same image overexposed to visualize the small viral dots (arrows) present in the nucleoplasm. RC: Replication Compartment; MA: multiple-acute; ML: multiple-latency; S: single; S+: single +. Scale bars represent 10 μm.

**Fig 2:**
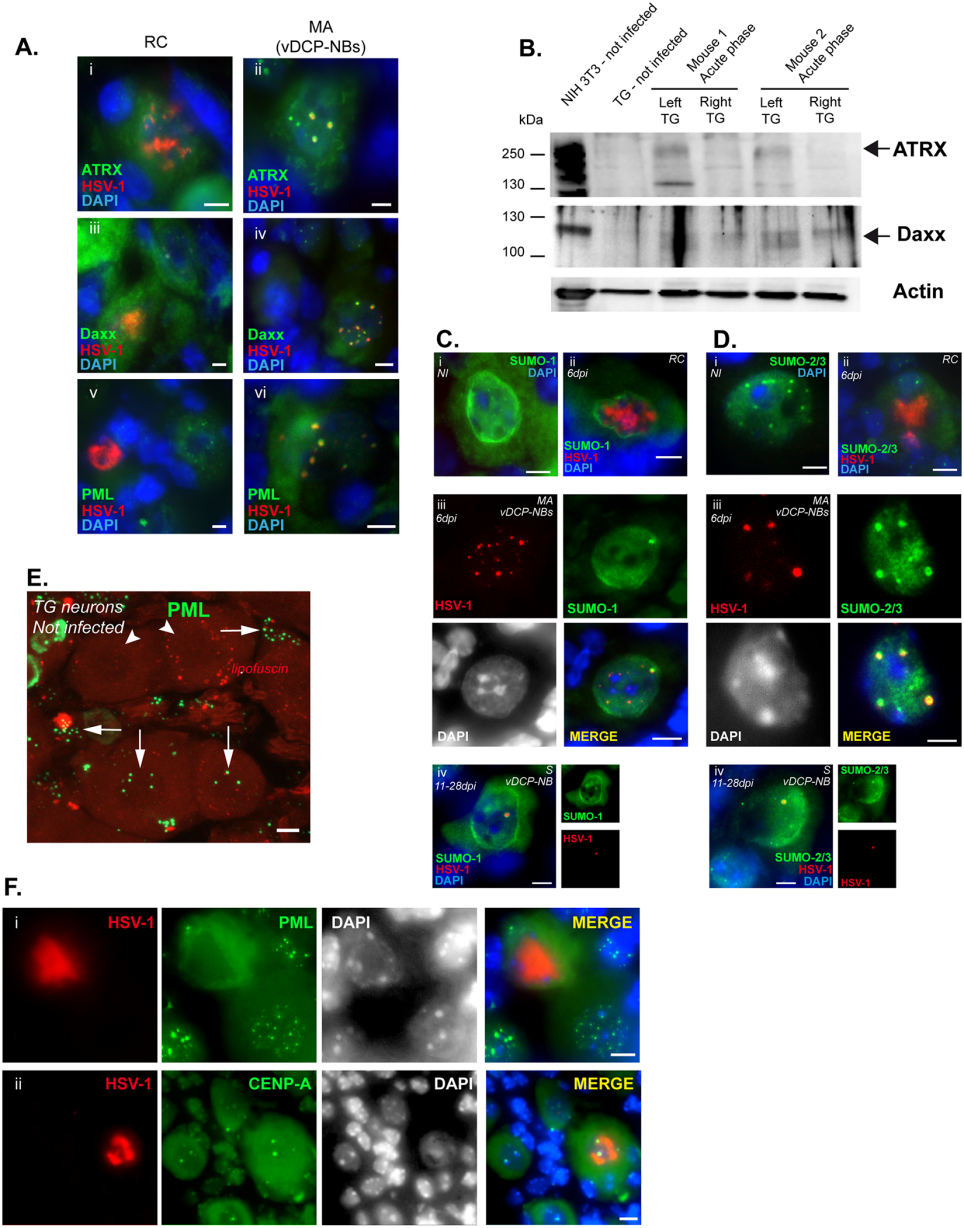
HSV-1 MA pattern corresponds to vDCP-NBs and contains SUMO proteins. (A) Immuno-DNA-FISH showing HSV-1 genomes (red), promyelocytic leukemia nuclear bodies (PML-NB)–associated proteins (green), and cellular chromatin (DAPI, blue). (B) WB of ATRX and Daxx in TG of uninfected or infected mice during acute infection. Infected (left TG) and not-infected (right TG) TGs of the same mouse (two mice) were harvested 6 dpi, and treated to perform WB. Actin is shown as a loading control. NIH3T3 is shown as a cellular control. (C) and (D) Immuno-DNA-FISH showing HSV-1 genomes (red), small ubiquitin modifier (SUMO) proteins (green), and cellular chromatin (DAPI, blue/grey). (C) SUMO-1 and (D) SUMO-2/3 detection in (i) non-infected neurons, (ii) RC-containing neurons and (iii, iv) MA/vDCP-NBs or S/vDCP-NB-containing neurons. (E) IF for detection of PML (green) in uninfected TG neurons and satellite cells. Arrows point out PML-NBs in neurons or satellite cells, arrowheads point out neurons without PML-NBs. (F) Immuno-DNA-FISH showing HSV-1 genomes (red, RC) and (i) PML or (ii) CENP-A (green), in neurons. DAPI staining (grey) shows nuclei. Scale bars represent 10 μm.

In a previous study, we described the distribution of viral genomes in the nucleus of latently infected mouse TG neurons (28 days post-infection, dpi). We found that two major patterns were detectable; i.e., “single” (hereafter S) and “multiple-latency” (hereafter ML). Neurons harboring those patterns differed in LATs expression, with S- and ML-containing neurons being negative and positive, respectively. These viral genome patterns are likely to be among the key features that determine which neurons sustain reactivation. It was thus essential to characterize the nuclear distribution of the viral genomes during the whole process of establishing latency. Mice were infected and TGs were harvested at fixed times (0, 4, 6, 8, 11, 14, 18, 22, and 28 dpi) after inoculation. At 6 dpi, two major viral genome patterns were observed, which we named “replication compartment” (RC) and “multiple-acute” (MA) (Fig 1Ai and 1Aii). Some RC-containing neurons clearly showed annexation of the interchromosomal space (Fig 1Ai), as described previously in cultured cells [48]. The MA was distinguishable from the ML pattern on the basis of the following structural and temporal observations: (i) viral genome spots in the MA pattern were often larger than those in the ML pattern; (ii) neurons with the MA pattern showed up to 10 spots per nucleus, whereas neurons with the ML pattern could contain up to 50 detectable viral genome spots; (iii) viral genomes in the MA pattern co-localized with PML (see Fig 2Avi in this study, and Fig. 5C in [47] for a more precise analysis), forming the previously described “viral DNA-containing PML-NBs (vDCP-NBs, up to 10 per infected neuron) [47], whereas in the ML pattern only one or two spots of viral genome co-localized with PML [47]; (iv) MA pattern is detectable during acute infection and mainly at 6 dpi, whereas ML pattern build up begins from 8 dpi and then persists until latency *per se* (28 dpi) (Fig 1B).

We analyzed the proportion of neurons with the various viral genome patterns during the whole establishment of latency period from 4 to 28 dpi. Data were collected from two to three mice and are presented as estimations within three percentage ranges (0 to 10%, between 10 and 25%, and > 25%). We could distinguish five major patterns: RC, MA, ML, 4-3-2 spots (4-3-2), and S-single+ (S+) (Fig 1B). RC were visible only during the early stages of acute infection (from 4 to 6–8 dpi), MA appeared from 6 dpi and persisted not beyond 11 dpi, and 4-3-2 was detectable only during a short period between 11 and 14 dpi; ML and S were the two major patterns observed during latency (28 dpi, see [47]) and started to build up from 6–8 dpi for the former and 8–11 dpi for the latter. Similar to S and MA, the 4-3-2 patterns corresponded to vDCP-NBs.

An intriguing observation was a change in the number of vDCP-NBs per infected neuron from 6 dpi (up to 10 per neuron) to 14–28 dpi (only 1 per neuron), with an intermediate situation consisting in the 4-3-2 pattern (11 to 14 dpi). These data suggested the possibility of fusion of the vDCP-NBs as the process of establishment of latency progressed. To investigate this possibility we used an *in vitro* model involving infection of human primary fibroblasts with a replication-defective HSV-1 mutant, in1374. This virus does not replicate at 38.5°C and forms vDCP-NBs in human primary fibroblasts and in neurons (see Figs S1A and 4B). Cells were harvested at 6 h to 7 dpi and processed for immuno-FISH to visualize viral genomes and PML. At 6 hpi the number of vDCP-NBs was 1 to 20/nucleus with an average of 5 and an average area of viral spots of 30.5 nm^2^ (minimum 1.8 nm^2^, maximum 120.6 nm^2^). The average number of vDCP-NBs/nucleus decreased over time to 1 to 6 vDCP-NBs/nucleus with an average of 2 at 7 dpi, and an average area of the viral spots of 116.9 nm^2^ (minimum 19.8 nm^2^, maximum 279 nm^2^) (S1B–S1D Fig). Given the experimental conditions used to perform cell infections (see Materials and Methods), it is unlikely that the decrease in viral spot number is due to loss of viral genomes over time due to multiple cell divisions. We counted the number of cells/well at the start and end of each experiment; the results indicated little cell division (S1E Fig). We then determined the total number of viral genomes by quantitative PCR at the start and end of each experiment to rule out an effect of viral genome degradation. Although, we observed a slight (but not significant) loss of viral genomes between 6 and 24 hpi (possibly due to limited cell division at the start of the experiment due to inertia of cells seeded 24 h before the infection), no significant loss of viral genomes was detected (S1F Fig, Student’s *t*-test). Data obtained *in vitro* on the amount and size of vDCP-NBs, combined with the *in vivo* observations, suggested that vDCP-NBs are dynamic structures probably capable of fusion as the establishment of latency progressed. Overall, the *in vivo* data anticipate changes in the viral genome patterns during the whole process of establishment of latency until the system achieves the physiological, cellular and molecular conditions that enable stable latency to be maintained.

We then determined the presence of HSV-1 genomes in cells other than neurons. TG from mice infected for 4 to 6 dpi were analyzed by immuno-FISH, and neurons were specifically labeled with an antibody recognizing neurofilaments. We detected viral genomes under the RC pattern in neurons and satellite cells (Fig 1Ci and 1Ciii), whereas MA was exclusively detected in neurons (Fig 1Cii). RC- but not MA (or ML, S2 Fig)-containing neurons were positive for ICP4 and ICP27, two of the major proteins of the lytic cycle (Fig 1Di–1Div). This confirmed that during acute infection, neurons positive and negative for productive infection-associated proteins harbored different HSV-1 genome patterns. Overall, these data emphasized the discrepancies in the viral genome nuclear distribution between infected neurons during the whole process of establishment of latency, and the link between these patterns and the capacity of the infected neuron to support a lytic cycle or latency. RC-containing neurons are likely to become productively infected, whereas MA-containing neurons are likely among those that will support latency.

### Viral genome patterns correlate with latency transcriptional program

One of the molecular characteristics of latency is a switch in the virus transcriptional program towards the quasi-exclusive expression of an abundant lncRNA known as LAT. We thus analyzed LAT expression in neurons containing the various viral genome patterns. We performed combined RNA/DNA FISH on TG harvested from acutely infected mice. RC-containing neurons were in their vast majority negative for LAT, with the exception of a few (< 1%) (Fig 1Ei and 1Eii). This was not unexpected, as previous studies reported that some latently infected neurons could experience an aborted lytic program [49,50]. MA and S-containing neurons were negative for LAT (Fig 1Eiii and 1Ev; lower neuron), with the exception of rare MA-containing neurons that contained discrete LAT signals juxtaposed to viral genomes (Fig 1Eiv). The only neurons frequently positive for LAT detection were those with the ML and S+ patterns (Fig 1Ev upper neuron and vi) as described previously [47]. Of note is that, with the exception of the few neurons positive for LAT at 4–8 dpi, LAT was readily detectable only from 10–14dpi.

### Viral genomes co-localize with PML-NB–associated proteins

We previously showed that the MA viral genome pattern co-localizes with, PML, Daxx and ATRX, three of the major constituents of the PML-NBs, thereby forming vDCP-NBs [47]. RC-containing neurons lacked the typical PML-NB staining for PML, Daxx and ATRX (Fig 2Ai, 2Aiii and 2Av), unlike those containing the MA pattern, which showed vDCP-NBs (Fig 2Aii, 2Aiv and 2Avi). We noticed an increase in both Daxx and ATRX signals in infected compared to uninfected TGs at 6 dpi (S3 Fig). We then performed WBs on whole TG extracts from uninfected and infected mice to analyze the signal of both proteins (Fig 2B). The model of virus inoculation used (upper left lip, see Materials and Methods) allows the mouse to be heavily infected in the left TG but not in the right TG. WBs were performed on the two TGs (left: infected, right: not infected) of two mice. Similar to what was previously observed for PML [47], we detected an increase in the amount of both proteins in the infected compared to the uninfected TGs. These data showed that during acute infection, and similarly to PML, the overall amount of Daxx and ATRX increased, probably as a result of the antiviral response mediated in the entire TG by type 1 interferons.

Small Ubiquitin MOdifier (SUMO) proteins are also major components of the PML-NBs and are involved in the intrinsic antiviral response against HSV-1 infection in cell cultures [23,51]. We analyzed the involvement of SUMOs in the control of virus infection in TG neurons, by co-detecting SUMOs and viral genomes during the whole process of establishment of latency. SUMO-1 and SUMO-2/3 were found in PML-NBs in uninfected neurons (Fig 2Ci and 2Di). In RC-containing neurons, similar to PML, Daxx, and ATRX, SUMO-1 and SUMO-2/3 did not show the punctate pattern characteristic of their presence in PML-NBs (Fig 2Cii and 2Dii). In MA-containing neurons, SUMO-1 was infrequently (< 20% of infected neurons) found co-localized with not more than one vDCP-NB (Fig 2Ciii), whereas SUMO-2/3 was frequently (> 50% of infected neurons) co-localized with all the vDCP-NBs (Fig 2Diii). Co-localization of SUMO-2/3 with vDCP-NBs persisted until 28 dpi, and SUMO-1 was found to be more systematically co-localized with vDCP-NBs in the S pattern from 14 dpi onwards (Fig 2Civ and 2Div). These data suggest the involvement of SUMO proteins in control of the incoming viral genomes, in accordance with their previously described intrinsic antiviral activity. However, in neurons, the activity and nuclear dynamics of SUMO-1 and SUMO-2/3 could differ with regard to their association with vDCP-NBs.

ICP0 is involved in the proteasomal degradation of several components of the PML-NBs, inducing the destabilization of these nuclear bodies. RC-containing neurons were consistently negative for the presence of PML-NBs. In these neurons, could ICP0 have induced destabilization of the PML-NBs, favoring the lytic cycle? Although we possess several ICP0 antibodies that have been used by us and others in immunocytochemistry, we have not detected an ICP0 signal within infected TG neurons by either IF or immuno-DNA FISH. Indeed, ICP0 in infected cell cultures shows a nuclear punctate pattern that is difficult to distinguish from the nonspecific signal in neurons from TG samples. An indirect way to analyze ICP0 activity in infected nuclei is to detect the disappearance of its cellular substrates. In addition to PML, the centromeric protein A (CENP-A) is another ICP0 substrate, and ICP0 efficiently induces proteasomal degradation of centromeric proteins in mouse cells [28]. TG samples from mice infected for 6 days were processed by immuno-DNA FISH to determine the fate of PML or CENP-A signals in infected neurons. All RC-containing neurons were negative for PML-NBs but positive for the CENP-A signal (Fig 2Fi and 2Fii). Several hypotheses arose from these data: (i) ICP0 is less efficient in inducing the degradation of centromeric proteins than PML in mouse neurons, (ii) ICP0 is synthesized in, but does not reach the nucleus of, infected neurons, (iii) ICP0 is not efficiently synthesized in neurons, (iv) some neurons lack PML-NBs and are more susceptible to lytic infection even in the absence of functional ICP0. Concerning the latter, IF analyses of TG samples from uninfected mice showed that not all neurons contained PML-NBs (Fig 2E). Moreover, a previous study suggested that ICP0 remained in the cytoplasm of HSV-1-infected TG neurons in culture [52]. These data do not exactly fit with our results (see S4Ci Fig), possibly due to the heterogeneity in the type of neurons found in a TG, combined with the use of different methods of purification of neurons in the two studies. Finally, a recent study demonstrated that a neuron-specific microRNA, miR-138, targets ICP0 mRNA, preventing ICP0 synthesis at least in cultured cells [53]. Therefore, our data, together with those of other laboratories suggested that during acute infection the interplay between cellular (including PML-NB-associated proteins) and viral factors is likely to determine the extent of virally-induced modification of the nuclear environment. Depending on the degree of modification, the lytic or latent transcriptional program will then be favored, leading to acquisition of the corresponding viral genome pattern.

### Characterization of PML-NB and viral protein synthesis in infected mouse primary TG neuron cultures

**Fig 3:**
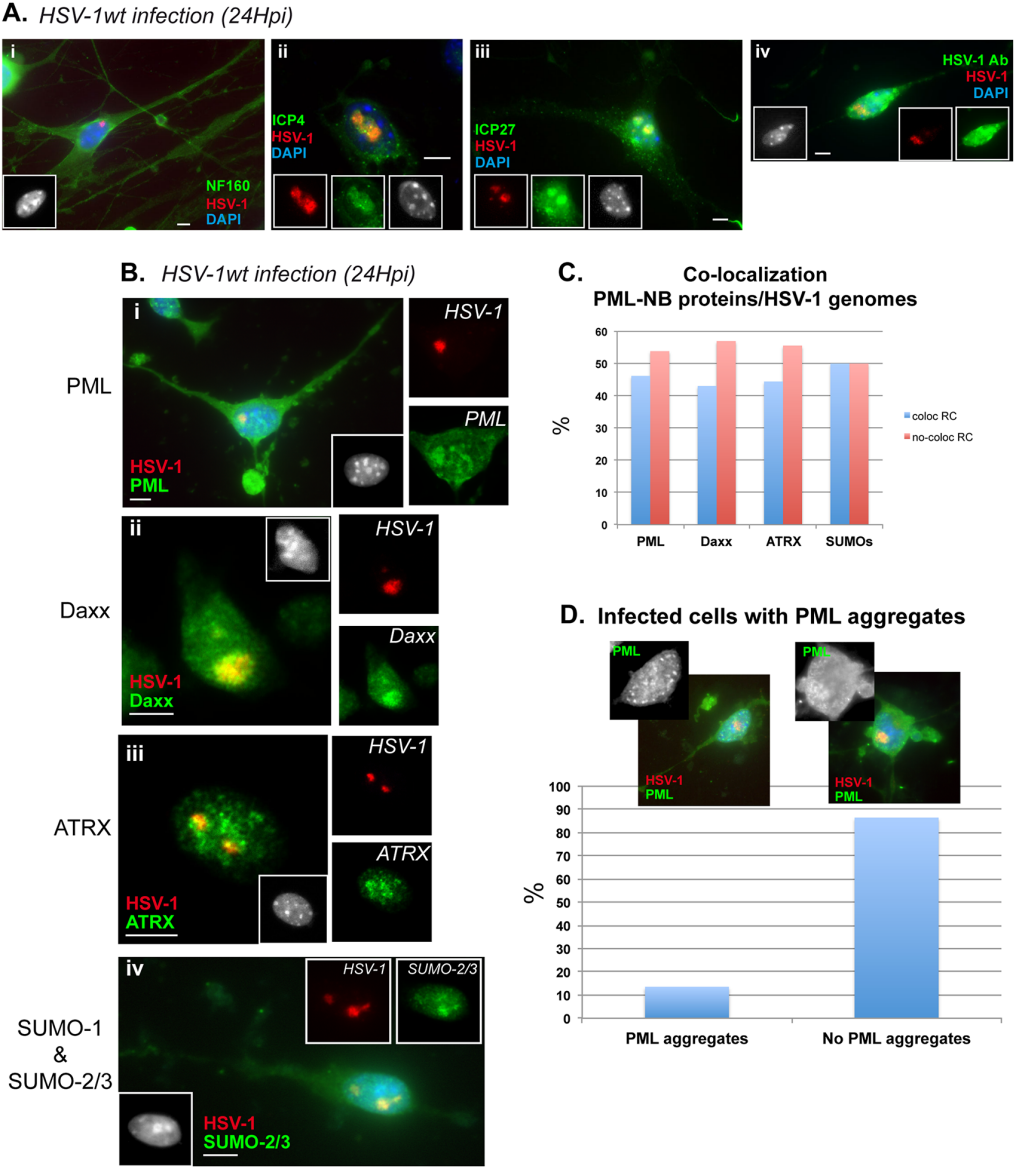
Behavior of PML-NB-associated proteins in HSV-1 infected cultured primary mouse TG neurons. (A) Immuno-FISH detection of neuronal or viral markers (green) and HSV-1 genomes (red) in RC-containing neurons. (i) neurofilaments, (ii) ICP4, (iii) ICP27, (iv) total HSV-1 proteins. (B) Immuno-FISH detection of PML-NB components (green) and HSV-1 genomes (red) in RC-containing neurons. (C) Quantification of infected neurons showing co-localization of HSV-1 genomes with PML-NB components. Fifty to 80 neurons were counted for each labeling. (D) Quantification of RC-containing infected neurons showing or not PML aggregates. Scale bars represent 10 μm.

To gain further insight into the cellular and molecular features that favor acquisition of the various viral genome patterns, especially vDCP-NBs and ML, which are associated with the latency process, we established mouse primary TG neuron cultures (S4A Fig). We first characterized the PML-NBs in the neurons and found that Daxx, ATRX, SUMOs and PML proteins were detectable in the nuclear bodies in most neurons (S4Bi–S4Biv Fig). We then infected the neuron cultures with HSV-1wt 24H to detect the synthesis of viral IE proteins involved in the onset of lytic infection, such as ICP0, ICP4 and ICP27 (S4Ci–S4Civ Fig). All proteins could be found in the nucleus of the infected neurons. We then performed immuno-FISH on similarly infected neurons to detect simultaneously viral genomes and neuronal or viral markers. As expected, all infected neurons showed RC, and expressed lytic viral proteins as exemplified by specific detection of ICP4, ICP27, or viral proteins (Fig 3Ai–3Aiv). ICP0 could not be detected in these experiments because none of the antibody that we usually use was suitable for our immuno-FISH protocol. We then analyzed the fate of PML-NBs and associated proteins. The majority (about 88%) of infected neurons did not show PML-NBs although some (about 12%) contained PML dots characteristic of PML-NBs (Fig 3D). About half of these infected neurons (Fig 3C) showed co-localization of PML-NB-associated proteins with RC (Fig 3B). This was not unexpected, as previous studies have described the presence of PML in RC of HSV-1-infected cells [54,55]. Because ICP0 is directly involved in the destabilization of PML-NBs in infected non-neuronal cells, we performed infections with a deletion virus unable to express ICP0. Neurons were infected for 24 h with the dl1403 mutant virus and PML-NBs were analyzed. In infected neurons, PML was found both in dots and co-localized with the RC (S5Ai Fig). Dots of PML were frequently located at the edge of and all around the RC (S5Aii Fig). SUMO proteins remained co-localized with PML in the PML dots (S5Avi Fig); however Daxx and ATRX were absent from the remaining PML dots (S5Aiii to S5Av Fig). In infected mice, a virus with deletion of the thymidine kinase (TK) gene is able to replicate more or less efficiently at the site of inoculation but its replication in TG neurons is severely impaired [56,57]. To determine if the absence of TK alone could explain the formation of vDCP-NBs in infected neurons, we infected neurons with a TK mutant HSV-1 virus 17/tBTK^-^ and analyzed the viral genome patterns at 48 hpi. Neurons with RC and without PML-NBs were exclusively observed, likely as a result of ICP0 expression (S5Bi to S5Biv Fig). Taken together, these data showed that, under our experimental conditions, which were compatible with the detection of viral genomes by FISH, if neurons are infected through the cell body and not through the axon as in natural infections, the balance between pro- and antiviral features favors the onset of lytic infection and formation of RC.

### Absence of functional ICP4 and ICP0 is required for the formation of vDCP-NBs

**Fig 4:**
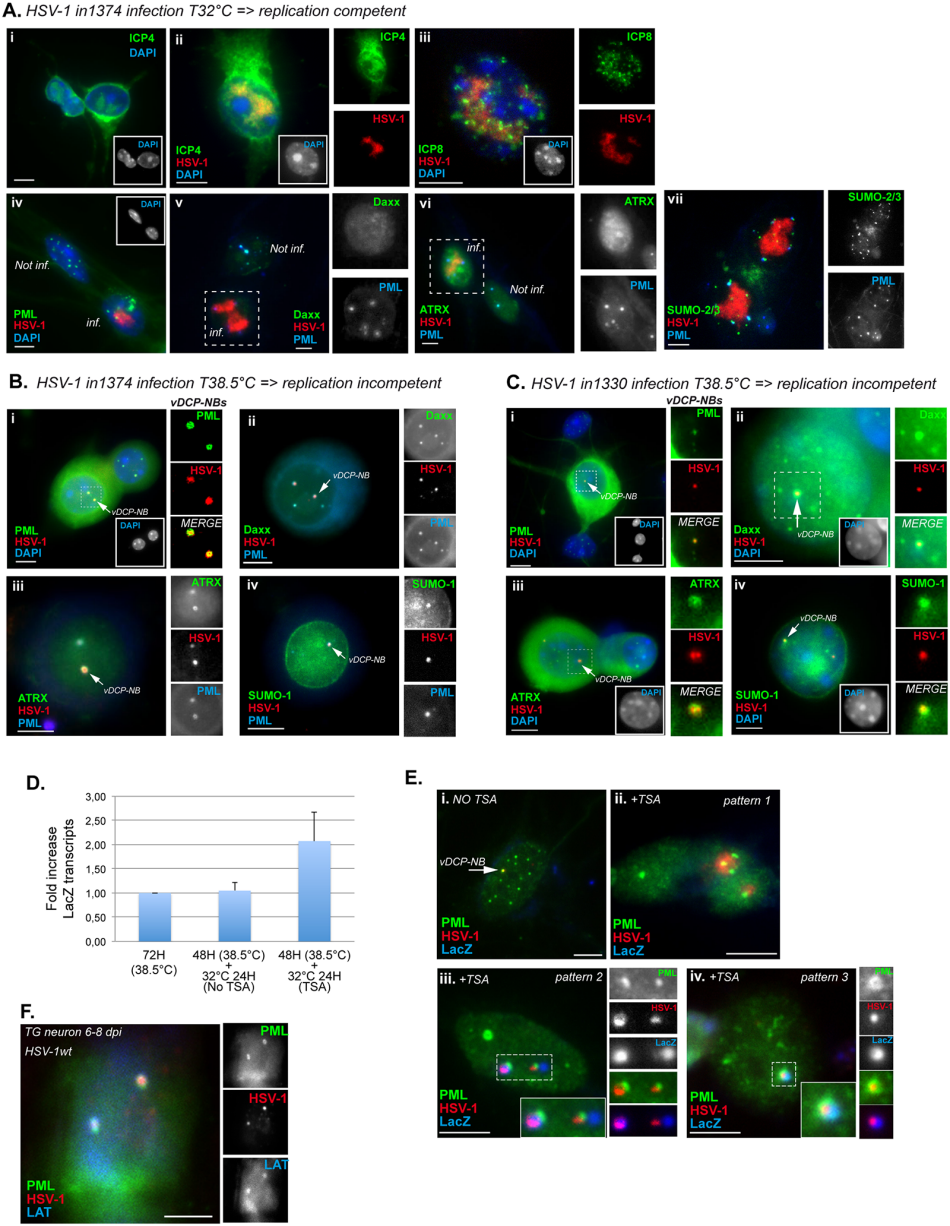
vDCP-NBs are formed in the absence of functional ICP4 and ICP0 in cultured primary mouse TG neurons, and can be associated with viral transcription. (A) Neurons were infected with *in*1374 at 32°C for 24 h. (i) IF detection of ICP4, (ii and iii) immuno-FISH for detection of ICP4 or ICP8 and HSV-1 genome, and (iv–vii) immuno-FISH for the detection of PML, Daxx, ATRX or SUMO-2/3 and the HSV-1 genome. (B) and (C) Neurons were infected at 38.5°C for 48 h with *in*1374 and *in*1330, respectively. (i–iv) immuno-FISH for the detection of PML, Daxx, ATRX or SUMO-1 and the HSV-1 genome. (D) RT-qPCR for the detection of *LacZ* transcripts in *in*1374 infected neurons treated or not with trichostatin A (TSA). Results show means (± SD) of two independent experiments. (E) RNA-DNA FISH combined with IF for detection of *LacZ* transcripts (blue), HSV-1 genomes (red), and PML (green) in *in*1374 infected neurons treated or not with TSA. Three different patterns are shown. (F) RNA-DNA FISH combined with IF for detection of LAT transcripts (blue), HSV-1 genomes (red), and PML (green) in mouse TG neurons at 6 to 8 dpi. Scale bars represent 10 μm.

Studies performed in infected mice using HSV-1 or pseudorabies virus (PRV), another neurotropic herpesvirus infecting pigs, showed that VP16, which is present in the virion tegument, is inefficiently transported through the axon to the cell body of infected neurons [58,59]. Another study described the distinct regulation of the VP16 promoter (normally a late promoter) in TG neurons, which could be activated early after infection by neuron-specific factors [60]. The stochastic activation of the VP16 promoter in neurons would thus enable the early synthesis of VP16 during acute infection as well as reactivation from latency. This would favor the entry of the virus to the lytic cycle through the activation of IE genes, including ICP4 and ICP0. In that context, the efficiency of the PML-NB–associated intrinsic antiviral response is likely to be inversely proportional to the synthesis of VP16, ICP4 and ICP0, and influence the acquisition of the latency viral genome pattern. To test this hypothesis, we infected neurons with the temperature-sensitive virus, *in*1374, which inefficiently expressed functional ICP4 at the restrictive temperature of 38.5°C, and lacks functional VP16 and ICP0 due to an insertion of 12 nt in the transactivation domain of the former and to deletion of part of the RING finger of the latter [61–63]. Neurons were first infected at the permissive temperature of 32°C. As expected, the virus showed the same features as HSV-1wt in terms of the formation of RC co-localized with ICP4 (Fig 4Ai and 4Aii), and ICP8, a subunit of the viral DNA replication complex (Fig 4Aiii). PML, Daxx, ATRX, and SUMO signals were similar to those obtained in neurons infected with the ICP0 mutant dl1403, with PML aggregates surrounding the RC and co-localizing with SUMOs, and Daxx and ATRX disappearing from these structures (Figs 4Aiv–4Avii and S3A). We then infected neurons at the restrictive temperature to inactivate ICP4. Under these conditions, viral genomes showed a different pattern and were detected as spots in the nucleus of infected neurons (Fig 4B). The co-detection of PML, Daxx, ATRX and SUMOs showed perfect co-localization of all proteins with the viral genomes, resulting in formation of structures reminiscent of vDCP-NBs (Fig 4Bi–4Biv). Infection with *in*1330 virus, which contains and expresses a functional VP16 (and hence expresses functional IE proteins ICP27, 22 and 47), also led to the formation of vDCP-NBs at 38.5°C (Fig 4Ci–4Civ). Infections with the *ts*K virus, which is the parental virus that expresses tsICP4 at 38.5°C and contains functional VP16 and ICP0, exhibited mainly RC (S6 Fig). This is possibly explained by the expression of a fraction of functional ICP4 at 38.5°C that under our experimental conditions of infection of neurons is sufficient to activate the lytic transcriptional program.

If the formation of vDCP-NBs is the default viral genome pattern for a virus unable to replicate in neurons, than infection of mice with a virus deficient in replication in neurons should lead to the exclusive formation of vDCP-NBs. Mice were infected with the TKDM21 mutant virus, which can replicate at the inoculation site, but is unable to replicate in neurons due to a deletion in the TK gene [56,57,64] (S7A and S7B Fig). Mice at 6 dpi were sacrificed and immuno-FISH was performed on TG samples to detect HSV-1 genomes and PML. Complete TGs of four mice were analyzed, and few neurons (48 in total) were detected with a positive signal for the viral genome. The small number of positive neurons may be due to weak replication of the virus in the lip (S7A Fig). This small number of positive neurons hampers precise analysis. However, none of the thousands of neurons analyzed in the four mice showed an RC pattern. All positive neurons showed vDCP-NBs comprising 35 (73%) with a “single” (one spot) pattern, 8 (17%) with two spots, 3 with three spots (6%), and 2 with four spots (4%) (S7C Fig). These data, although obtained from few detectable infected neurons, tend to confirm those obtained in the cultured neurons and suggest that the inability of a virus to start replication in neurons will automatically lead to the formation of vDCP-NBs probably due to the additional absence of ICP0 synthesis due to cellular miR control [53]. Overall, these data demonstrated that the formation of vDCP-NBs resulted from both the failure of the virus to start the lytic program due to the inefficient synthesis of ICP4 (and thus to undergo replication), and the absence of functional ICP0. Although the presence of functional VP16 *per se* did not directly impact the formation of vDCP-NBs, its stochastic synthesis in neurons during acute infection likely increases the probability of ICP4 synthesis, and thus the start of the lytic program and the formation of RC.

### HSV-1 genomes present in vDCP-NBs can be transcriptionally reactivated

Our previous data showed that latently infected neurons containing vDCP-NBs were deficient in the expression of the 2 kb LAT, and that viral genomes trapped in the vDCP-NBs were unable to synthesize the primary 8.3 kb LAT transcript [47]. These data raise the question of whether the genomes in the vDCP-NBs are permanently silenced, or if they retain the capacity to resume transcription following exposure to stress that could affect the vDCP-NBs. *In*1374 contains a HCMV-*lacZ* cassette whose transcription is shut down upon infection of human fibroblasts at 38.5°C and resumes upon treatment with the histone deacetylase inhibitor, trichostatin A (TSA) [65,66]. Similarly, mouse primary TG neurons quiescently infected with a non-replicative HSV-1 virus containing a pCMV-GFP transgene were shown to resume GFP expression upon the addition of TSA [67]. Neurons infected with *in*1374 for 3 days were treated or not with 2 μM TSA for 24 h at 32°C and RT-qPCR was first performed to quantify *LacZ* re-expression under these experimental conditions (Fig 4D). Dual RNA-DNA FISH was then performed to detect *LacZ* transcripts and HSV-1 genomes. Without TSA, viral genomes in the vDCP-NBs showed no sign of *LacZ* transcription (Fig 4Ei). Addition of TSA led to the observation of three further patterns: (i) *LacZ*-negative RC-like structures in close proximity to PML-NBs (Fig 4Eii, pattern 1); (ii) RC-like structures juxtaposed with *LacZ* and PML signals (Fig 4Eiii, pattern 2); and (iii) vDCP-NBs containing LacZ signal (Fig 4Eiv, pattern 3). To determine whether PML-NBs juxtaposed to stress-induced RC are missing Daxx and/or ATRX, similar to the HSV-1 dl1403 (ICP0-)-infected cultured neurons showing RC (see S5A Fig), we analyzed Daxx and ATRX behavior in RC-containing neurons. First, we confirmed that TSA treatment alone did not affect the localization of Daxx and ATRX at the PML-NBs in uninfected neurons (S8A Fig). Unlike PML, the majority of the stress-induced RCs did not juxtapose with Daxx or ATRX in spots, which suggested that the two proteins leave the RC-associated PML-NBs (S8B and S8C Fig). However, some neurons exhibited stress-induced RC in the vicinity of Daxx or ATRX spots, albeit with diffuse Daxx and ATRX signals throughout the nucleoplasm. Moreover, some neurons showed a Daxx signal co-localized with RCs. The two latter most likely reflect transitory situations before the complete disappearance of Daxx and ATRX from RC-associated PML-NBs. These data suggest that upon transcriptional reactivation leading to HSV-1 replication, PML remains in spots juxtaposed to the RC whereas Daxx and ATRX are more labile and tend to leave the RC-associated PML-NB. Daxx and ATRX behavior is in accordance with their previously described mutual contribution to intrinsic antiviral resistance to HSV-1 infection [34].

PML-associated pattern 3 (Fig 4Eiv) was reminiscent of the MA genome-associated discrete LAT signal reported previously (see Fig 1Eiv). TG neurons from 6 to 8 days HSV-1wt–infected mice were analyzed for the expression of LAT together with the detection of viral genomes and PML protein. Rare neurons were indeed positive for a discrete LAT signal associated with vDCP-NBs (Fig 4F). Together, these data showed that: (i) a virus contained in a vDCP-NB is unlikely to be definitively silenced provided that a stimulus sufficient to modify the transcriptional equilibrium and/or the PML-NBs dynamic is applied to the neuron; (ii) at least during the first stages of establishment of latency in mice (6–8 dpi), viruses associated with vDCP-NBs could show some transcription of LAT before being completely silenced during latency *per se* (28–30 dpi). A previous study performed in cultured cells showed that viral genomes juxtaposed to the PML-NBs were more prone to initiate replication [68]. Other studies suggested that HSV-1 transcription is more likely to occur in the vicinity of PML-NBs [69,70]. Our data, together with those of other groups, show that PML-NBs could have a dual role in viral infection; on the one hand, the capacity to silence incoming viral DNA, and on the other hand, and following appropriate stimuli, to serve as a nuclear platform for virus reactivation, although during this event the protein content of PML-NBs is likely modified in terms of Daxx and ATRX leaving the nuclear body.

### Type 1 interferon favors acquisition of the ML pattern in infected neurons

**Fig 5:**
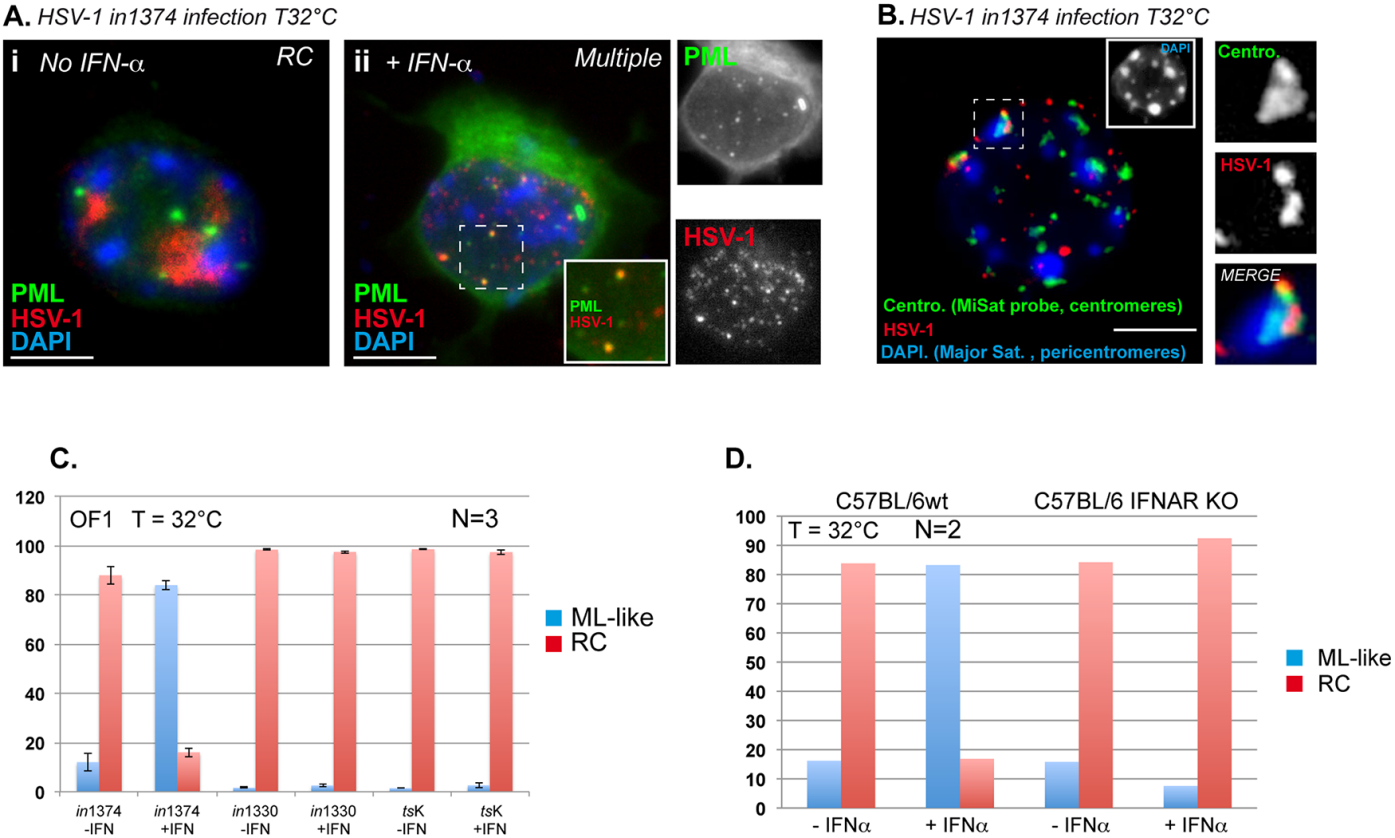
Type 1 IFNα induces an ML-like pattern in infected cultured primary mouse TG neurons. (A) Immuno-FISH for the detection of PML and HSV-1 genomes in cells not treated (i) or treated (ii) with IFNα (1000 IU/mL). (B) Double DNA FISH for the detection of centromeric minor satellite sequences and HSV-1 genomes in cells treated with IFNα (1000 IU/mL). (C) Quantification of ML-like and RC patterns in neurons (three independent experiments) infected with *in*1374, *in*1330, or *ts*K for 24 h in the absence or presence of IFNα (1000 IU/mL). Results show means (± SD). (D) Quantification of ML-like and RC patterns in neurons from C57BL/6 wt or IFNAR KO mice, and infected by *in*1374 for 24 h in the absence or presence of IFNα (1000 IU/mL). Means of two independent experiments are shown.

Type 1 IFNs are produced very early upon alphaherpesvirus infection, which limits virus replication and spread both *in vitro* and *in vivo* [71–74]. IFNα was previously shown to induce a quiescent state of HSV-1 that resembles latency in cultured primary porcine TG neurons [75]. Given the changes in the viral genome patterns observed during acute infection (see Fig 1), we anticipated that type 1 IFN could take part in those changes by preventing the onset of lytic infection even in the presence of functional ICP4, provided that functional VP16 and ICP0 are missing. We infected neurons with *in*1374 at 32°C in the presence or absence of IFNα. Without IFNα, only RC-containing neurons were observed (Fig 5Ai). Treatment of neurons with IFNα decreased the number of neurons showing the RC pattern, and induced the formation of multiple spots in the nucleus, some of which co-localized with PML-NBs or centromeres, a pattern reminiscent of the ML pattern *in vivo* (Fig 5Aii and 5B) [47]. Quantification of the effect of IFNα on pattern acquisition (Fig 5C) showed that without IFNα treatment infection with *in*1374 led to the formation of RC in ~91 (±3.5)% of neurons. IFNα treatment favored the ML-like pattern, with 82 (±1.7)% of neurons showing this pattern. Infection with *in*1330 or *ts*K induced the formation of RC in almost all infected neurons (> 98%), irrespective of IFNα addition, consistent with the essential contribution of VP16 and ICP0 to the onset of the lytic cycle. Type I IFNs share the same receptor, type I interferon receptor (IFNAR) [76]. To gain insight into the impact of the IFN signaling pathway on viral genome pattern acquisition, we infected TG neurons harvested from IFNAR KO mice with *in*1374 at 32°C. Irrespective of treatment with IFNα, nearly all infected neurons (> 98%, two experiments) showed the formation of RC (Fig 5D), whereas infection of wt C57BL/6 neurons yielded results similar to those of OF1-infected neurons. These data confirmed that the type I IFN signaling pathway plays a major role in viral genome pattern acquisition, and favors formation of the ML-like pattern provided that functional VP16 and ICP0 are not produced in infected neurons.

### HSV-1 genomes in latently infected human TG neurons co-localize with PML protein

**Fig 6:**
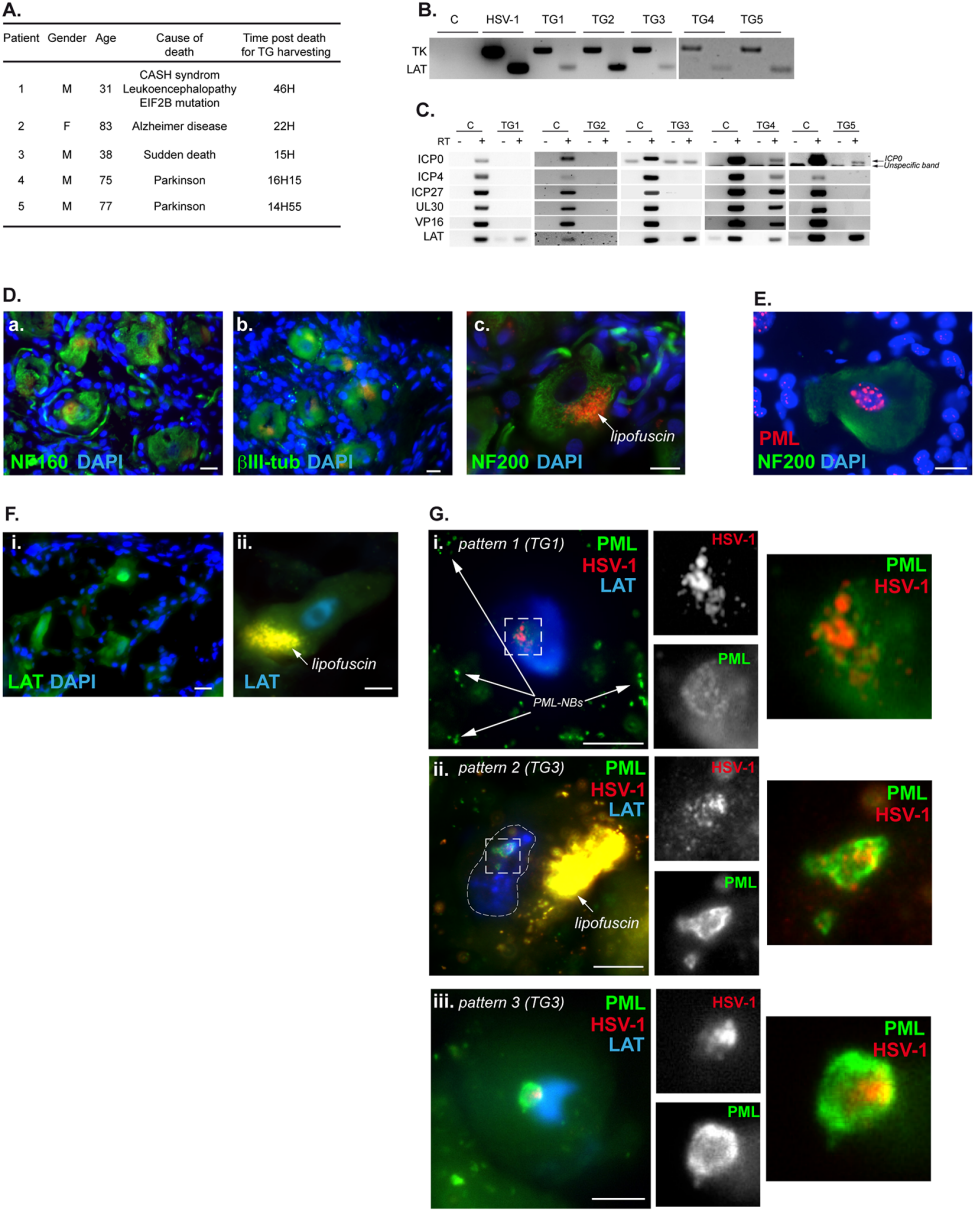
Detection of HSV-1 genomes in neurons from human TGs. (A) Table showing the characteristics of the five patients from which TGs were analyzed. (B) PCR for the detection of HSV-1 genomes in the five TGs harvested from patient 1 to 5. TK and LAT loci were detected. The lane HSV-1 indicates viral genomes detection in infected cells used as a positive control. C is the negative control issued from uninfected cells. (C) RT-PCR for detection of several lytic transcripts and LAT in the five human TGs. C: RNA from controlled infected cells (for ICP0, 4, 27, UL30 and VP16 detection) or mouse TG (for LAT detection). RT is for Reverse Transcription. “-”and “+” indicate samples without or with reverse transcription, respectively. (D) IF for the detection of three neurofilament markers (NF160, NF200 and ßIII-tub). (E) IF for the detection of PML and neurofilaments. (F) RNA FISH for LAT detection using tyramide 488 (i, green) or tyramide 350 (ii, blue). (G) RNA-DNA FISH combined with IF for the detection of LAT transcripts (blue), HSV-1 genomes (red), and PML (green). Three different PML patterns are shown. The PML signal in non-neuronal cells is shown in (i). Scale bars represent 10 μm.

The formation of vDCP-NBs is an important hallmark of HSV-1 latency in mice and likely highlights a close inter-connection at the molecular level between the intrinsic antiviral activity of PML-NBs and the latent viral genomes. To investigate this association in the context of HSV-1 latency in human, we performed analyses of human TGs. The left and right TGs of five patients (Fig 6A) were collected and then processed to analyze the presence of HSV-1 genomes by PCR and possible reactivation by RT-PCR of viral transcripts, and also by immuno-FISH. PCR data showed that all TGs were positive for viral genomes (Fig 6B), and one patient was reactivating HSV-1 at the time of collection (Fig 6C). To avoid any misinterpretation due to reactivation, we further analyzed in priority the TGs that did not show any signs of reactivation at the molecular level. We performed IF using several neuronal markers to correlate the structural analysis of the cells with biochemical markers specific to neurons. Neurons appeared as large cells, usually containing a bright cluster of cytoplasmic autofluorescence due to lipofuscin, and with markedly fainter nuclear DAPI staining than that of satellite cells (Fig 6Di–6Diii). PML-NBs appeared in neurons as large nucleoplasmic aggregates of PML protein (Fig 6E). Detection of the 2 kb LAT showed bright staining throughout the nucleoplasm, irrespective of the probe and stain used (Fig 6F). Samples from TGs were subjected to simultaneous detection of LAT, HSV-1 genomes and PML. Several observations were made in neurons positive for HSV-1 DNA (Fig 6Gi–6Giii): (i) infected neurons unequivocally showed systematic disappearance of the large aggregates of PML-NBs observed in uninfected neurons; (ii) observations at high magnification showed that PML staining was reorganized under a single clustered nuclear signal, most of the time forming a large “doughnut-shaped” structure; (iii) viral genomes were detected as multiple spots clustered in a discrete region of the nucleoplasm; (iv) the clusters of viral genomes constantly co-localized with the PML clusters, and when PML was under the “doughnut-shaped” structure, HSV-1 genomes were localized inside those structures; and (v) all neurons positive for HSV-1 genomes were also positive for LAT. Overall, these data confirmed the close proximity of viral genomes and PML during latency in human TG neurons, and the occasional presence of vDCP-NB-like structures.

## Discussion

The interaction of chromosomal loci with their nuclear environment affects the transcriptional activity of particular genes [77]. Nuclear architecture is thus likely to greatly influence the fate of infection with nuclear-replicating viruses, such as herpesviruses. In this study, we demonstrated that the interaction of latent HSV-1 genomes with the nuclear environment is impacted by the activity of cellular components, such as PML-NBs and type I IFNs, but also by viral features such as the ability of the virus to enter the lytic cycle and to express ICP0. During establishment of HSV-1 latency in the mouse model, viral genomes adopt several nuclear patterns before reaching the two main patterns that are found during stable latency. The nuclear distribution of viral genomes changes greatly until 14 dpi, and stabilizes thereafter. This is in agreement with previous reports of extinction of lytic gene expression, acquisition of chromatin markers, and expression of LAT, which are major molecular features of HSV-1 latency and usually evident by 14 dpi [78–80]. This indicates that the battle between the virus and the infected neurons involves multiple changes in the interaction between the viral genomes and the nuclear environment until reaching a stable situation suitable for both the virus and the host cell.

The RC pattern was found in neurons and non-neuronal cells, whereas vDCP-NBs were found only in neurons. The expression of lytic proteins in infected neurons during acute infection was detectable only in RC-containing neurons. These data, combined with the observation that vDCP-NBs are nuclear structures found during the whole process of establishment of latency until latency *per se*, suggest that vDCP-NBs play a major role in pushing the virus towards latency. To that extent, the presence in the vDCP-NBs of SUMO proteins, which were shown in cultured cells to participate in intrinsic antiviral resistance to HSV-1 infection [23], strengthens the idea that PML-NBs in general and vDCP-NBs in particular are nuclear relays of the cellular intrinsic antiviral response to HSV-1.

Studies performed *in vivo* and *in vitro* showed that VP16 expression likely plays a major role in the onset of the lytic program in neurons [60], and a neuron-specific microRNA, miR-138, targets ICP0 mRNA to prevent its synthesis [53]. Therefore, during the initial infection of neurons, the concomitant absence (or reduced amount below a threshold) of VP16 and ICP0 probably prevents the virus from entering the lytic cycle. Cultured neurons infected with a virus that is unable to express a functional ICP4, and which concomitantly does not express functional ICP0, mimicked the infectious process that *in vivo* leads to the formation of vDCP-NBs. This demonstrates that as long as the virus is unable to activate the lytic program due to the absence of functional ICP4 (probably linked to the absence of VP16 *in vivo*), then the only other viral feature required for the formation of vDCP-NBs is the absence of functional ICP0.

Besides the formation of vDCP-NBs, another hallmark of the nuclear distribution of HSV-1 latent genomes is the ML pattern. The ML pattern-containing neurons accumulated in *in vivo*-infected TGs with a slight delay compared to the vDCP-NBs. In cultured neurons, type I IFNα induced the formation of an ML-like pattern in a non-functional VP16 and ICP0 context. The type I IFN pathway was essential for acquisition of the ML-like pattern because infection of cultured TG neurons prepared from IFNAR KO mice did not result in formation of the ML-like pattern in the presence of IFNα. Two lines of evidences suggest that the ML-like pattern mimics that *in vivo*; i.e., (i) a subset of viral genomes in the ML-like pattern co-localized with PML, and (ii) some viral genomes co-localized with centromeres. Previous studies showed that the tegument protein VP16 was inefficiently transported from the axon termini to the cell body, which precluded the initiation of lytic infection in neurons [58,59]. However, a subset of neurons supports lytic infection during the early stages of acute infection of mouse TG. This could be due to a change in the kinetics of VP16 expression in neurons whose promoter could acquire IE-like activity in some neurons due to its activation by an as-yet-unknown neuron-specific feature [60]. The absence of VP16 axonal transport, the stochastic regulation of its promoter, and the absence of ICP0 synthesis due to miR-138 activity are likely to lead to a nuclear environment that would favor the establishment of latency through the formation of vDCP-NBs and/or ML patterns, depending on the type I IFN signaling context of the infected neuron. Type I IFNs (IFNα and ß) are major actors in the interplay between the virus, the cell, and the immune response [81]. The IFN response was shown to build up within the infected TG during establishment of latency in mice by both autocrine and paracrine signaling pathways [72]. Previous reports suggested that TGs of infected mice could sustain several waves of virus infection from the site of inoculation at the periphery by means of a positive inter-site “feedback loop” [82]. This suggests that the immune environment of the TG in general, and the neurons in particular, is likely to be different between the first wave of infection and those following. It is thus likely that a virus entering a neuron from a second wave of infection will face a different antiviral environment more prepared to face the virus infection, especially through enhancement of IFN-associated innate immunity. This will inevitably lead to some molecular changes in the nucleus, and the viral genome ML pattern acquisition might reflect these nuclear changes. It is worth mentioning that two of the major components of the PML-NBs, PML and Sp100, are encoded by interferon-stimulated genes (ISGs), which leads to an overall increase in the protein levels following IFN stimulation. Our previous and present data showed that PML, Daxx and ATRX levels increased in the TG during acute infection [47]. Given that PML in the PML-NBs represents only 10% of the total nuclear PML [83], the increase in PML by type I IFN stimulation is likely to increase the “free” pool of PML in the nucleoplasm, promoting its repressor-associated activity. Moreover, the overall increase in Daxx and ATRX, two chaperones of histone H3.3 [84], might result in changes in the chromatinization of the viral genomes, which could be linked to changes in their nuclear distribution. IFNα has also been shown to increase HDACs activity, which favors the acquisition of repressive chromatin marks [85]. It is interesting to note that facultative heterochromatin repressive marks associated with latent viral genomes begin to accumulate on the viral genomes by 7 dpi during acute infection in a mouse ocular model of infection [80]. This roughly corresponds to the time at which ML pattern-containing neurons start to appear in our lip model (Fig 1B). Therefore, ML pattern formation might be tightly linked to the acquisition of specific chromatin-associated marks. We are currently investigating this aspect of viral genome dynamics.

HSV-1 latency, rather than being an inert situation, is a dynamic equilibrium likely generated by multiple attempts by the virus to complete full reactivation, which is repressed most of the time by tight control by the innate and adaptive immune responses [86–89]. Humans latently infected by HSV-1 undergo multiple spontaneous symptomatic, asymptomatic, and aborted reactivations that could be restricted at different stages of the reactivation process. Despite the differences in the physiology and history of infection between a several-year latently infected human and few-week infected mice, we observed a close spatial overlap between viral genome and PML protein signals in HSV-1–infected human TG neurons, similar to what is observed in mouse TG neurons. Viral genomes were detected as multiple spots grouped in a restricted area of the nucleus. PML-NBs systematically disappeared in infected neurons and PML protein accumulated in the same areas as the viral genomes, eventually forming structures reminiscent of vDCP-NBs. These data show that HSV-1 latency/reactivation in human TG neurons is likely to be closely associated with PML protein and PML-NBs activity.

Overall, this study describes the nuclear architecture and nuclear distribution of viral genomes as major determinants of HSV-1 latency. It confirms the close interrelation between PML-NBs and HSV-1 genomes in the establishment of latency through the formation of vDCP-NBs. Finally, it confirms that the spatial organization of HSV-1 genomes and PML is conserved in latently infected neurons in human TG, which indicates PML-NBs to be major HSV-1 genome interactants during latency and probably reactivation.

## Materials and Methods

### Ethics statement

All procedures involving experimental animals conformed to the ethical standards of the Association for Research in Vision and Ophthalmology (ARVO) Statement for the use of animals in research, and were approved by the local Ethics Committee of the Institute for Integrative Biology of the Cell (I2BC) and the Ethics Committee for Animal Experimentation (CEEA) 59 (Paris I) under the number 2012–0047 and in accordance with European Community Council Directive 86/609/EEC. All animals received unlimited access to food and water.

Human biological samples and associated data were obtained from Cardiobiotec Biobank (CRB-HCL Hospices Civils de Lyon BB-0033-00046). All tissue samples were obtained according to French ethics regulations (specifically, informed consent was obtained from patients for all samples). Cardiobiotec is authorized by the French Ministry of Social Affairs and Health (DC2011-1437), with transfer authorization AC 2013–1867.

### Virus strains, mice and virus inoculation: Primary mouse TG neuron cultures, cells

The HSV-1 SC16 wild-type (wt) and thymidine kinase (TK) mutant (TKDM21) strains were used for mouse infections and have been characterized previously [64,90]. HSV-1 17 *syn* + (17+) wt and mutant strains were used for infections of primary mouse TG neuron cultures. HSV-1 mutants 17/tBTK^-^ and dl1403 are deleted in TK and ICP0 genes, respectively [82,91]. The HSV-1 mutant *ts*K expresses a temperature-sensitive variant of the major viral transcriptional activator ICP4 [92,93]. *In*1374 expresses a temperature-sensitive variant of the major viral transcriptional activator ICP4 [61], and is derived from *in*1312, a virus derived from the VP16 insertion mutant *in*1814 [62], which also carries a deletion/frameshift mutation in the ICP0 open reading frame [63] and contains an HCMV-*lacZ* reporter cassette inserted into the UL43 gene of *in*1312 [66]. Virus *in*1330 is a VP16 rescuant of *in*1312 [65]. All of these viruses have been used and described previously [65]. All HSV-1 strains were grown in BHK-21 cells (ATCC, CCL-10) and titrated in U2OS cells (ATCC, HTB-96). *Ts*K was grown and titrated at 31°C. Viruses derived from *in*1312 were grown and titrated at 31°C in the presence of 3 mM hexamethylene bisacetamide [94].

Mice were inoculated and TG processed as described previously [47,90,95,96]. Briefly, 6-week-old inbred female BALB/c mice (Janvier Labs) were inoculated with 10^6^ PFU of virus into the upper-left lip. Mice were sacrificed at the indicated times from 0 to 28 dpi. Frozen sections of mouse TG were prepared as described previously [47,96].

Primary mouse TG neuron cultures were established from BALB/c, OF1, or C57BL/6 wt (Janvier Labs) or IFNAR KO (The Jackson Laboratory) mice, following a procedure described previously [97]. Briefly, 6–8-week-old mice were sacrificed before TG removal. TG were incubated at 37°C for 20 min in papain (25 mg) (Worthington) reconstituted with 5 mL Neurobasal A medium (Invitrogen) and for 20 min in Hank’s balanced salt solution (HBSS) containing dispase (4.67 mg/mL) and collagenase (4 mg/mL) (Sigma) on a rotator, and mechanically dissociated. The cell suspension was layered twice on a five-step OptiPrep (Sigma) gradient, followed by centrifugation for 20 min at 800 *g*. The lower ends of the centrifuged gradient were transferred to a new tube and washed twice with Neurobasal A medium supplemented with 2% B27 supplement (Invitrogen) and 1% penicillin–streptomycin (PS). Cells were counted and plated on poly-D-lysine (Sigma)- and laminin (Sigma)-coated, eight-well chamber slides (Millipore) at a density of 8,000 cells per well. Neuronal cultures were maintained in complete neuronal medium, consisting of Neurobasal A medium supplemented with 2% B27 supplement, 1% PS, L-glutamine (500 μM), nerve growth factor (NGF; 50 ng/mL, Invitrogen), glial-cell-derived neurotrophic factor (GDNF; 50 ng/mL, PeproTech), and the mitotic inhibitors fluorodeoxyuridine (40 μM, Sigma) and aphidicolin (16.6 μg/mL, Sigma) for the first 3 days. The medium was then replaced with fresh medium without fluorodeoxyuridine and aphidicolin.

Primary human fibroblasts BJ cells (ATCC, CRL-2522) were grown in Dulbecco’s Modified Eagle’s Medium (DMEM) supplemented with 10% fetal bovine serum, L-glutamine (1% v/v), 10 IU/mL penicillin, and 100 mg/mL streptomycin. BJ cells stop their division by contact inhibition, therefore to limit their division, cells were seeded until confluence before being infected at a multiplicity of infection (m.o.i.) of 3, and maintained in 2% serum throughout the experiment.

### Virus titration assays in infected mice

Lips were biopsied at the region of virus inoculation (commissural region) immediately after the animals were euthanized, and TGs were harvested as described in the experimental procedures. Tissues were ground in microtubes containing 250μL of ice-cold PBS. Three rounds of freezing/thawing were applied using liquid nitrogen, and samples were centrifuged and supernatants stored at -80°C until use. Serial dilutions were used to titrate the virus on VERO cells (ATCC, CCL-81).

### DNA-FISH and immuno-DNA FISH

HSV-1 DNA FISH probes were Cy3 labeled by nick-translation. Cosmids 14, 28 and 56 [98] comprising a total of ~90 kb of the HSV-1 genome were labeled by nick-translation (Invitrogen) with dCTP-Cy3 (GE Healthcare), and stored in 100% formamide (Sigma-Aldrich). The DNA-FISH and immuno-DNA FISH procedures for TG sections have been described previously [47,96]. Briefly, frozen sections were thawed, rehydrated in 1x PBS and permeabilized in 0.5% Triton X-100. Heat based unmasking was performed in 100 mM citrate buffer, sections were post-fixed using a standard methanol/acetic acid procedure, and dried for 10 min at RT. DNA denaturation of section and probe was performed for 5 min at 80°C, and hybridization was carried out overnight at 37°C. Sections were washed 3 x 10 min in 2 x SSC and for 3 x 10 min in 0.2 x SSC at 37°C, and nuclei were stained with Hoechst 33258 (Invitrogen). All sections were mounted under coverslips using Vectashield mounting medium (Vector Laboratories) and stored at 4°C until observation.

For immuno-DNA FISH, frozen sections were treated as described for DNA-FISH up to the antigen-unmasking step. Tissues were then incubated for 24 h with the primary antibody. After three washes, secondary antibody was applied for 1 h. Following immunostaining, the tissues were post-fixed in 1% PFA, and DNA FISH was carried out from the methanol/acetic acid step onward.

The same procedures were used for infected neuronal cultures except that the cells were fixed in PFA 2% before permeabilization.

### Dual RNA/DNA FISH

RNA FISH probe labeling and RNA FISH procedures were performed as described previously [96]. Biotinylated single-strand LAT RNA probe was prepared by *in vitro* transcription (Ambion) using plasmid pSLAT-2 as a template (gift from S. Efstathiou, University of Cambridge, UK). Biotinylated *LacZ* probe was prepared from the pCMV-*LacZ* plasmid (Clontech) using the nick-translation procedure (Invitrogen). Frozen sections were treated as described for DNA FISH up to the antigen-unmasking step using solutions containing 2 mM of the RNAse inhibitor ribonucleoside vanadyl complex. The sections were pre-hybridized in 50% formamide/2 × SSC and hybridized overnight with 60 ng of RNA probe in a 50% formamide buffer at 65°C for LAT and 37°C for *LacZ*. Sections were washed in 50% formamide/2 × SSC at 65°C, and in 2 × SSC at room temperature. Detection was performed using streptavidin-HRP conjugate, followed by Tyramide Signal Amplification (TSA, Invitrogen) with an Alexa Fluor 350- or 488-conjugated substrate, according to the manufacturer’s guidelines. The DNA-FISH procedure was performed starting from the methanol/acetic acid post-fixation step.

### Western blotting

TGs were collected at 6 or 28 dpi and snap-frozen. Frozen tissues were ground, thawed in lysis buffer (10 mM Tris-EDTA, pH 8.0) containing a protease inhibitor cocktail, and briefly sonicated. Protein extracts were homogenized using QiaShredders (Qiagen). Protein concentration was estimated by the Bradford method. Extracted proteins were analyzed by Western blotting using appropriate antibodies.

### Antibodies

The following primary antibodies were used:

Mouse Mab anti-mouse PML (mAb3739; Millipore), anti-human PML (clone 5E10; Roel van Driel or clone PG-M3; Santa Cruz), anti-SUMO-1 (clone 5B12; MBL), anti-SUMO-2/3 (clone 1E7; MBL), anti-NF160 (Invitrogen), anti-ßIII tubulin (MAB1637; Millipore), anti-ICP0 (Mab11060), anti-ICP4 (clone 10F1; Virusys), anti-ICP27 (Virusys); rabbit Mab anti-SUMO-1 (clone Y299; Abcam), anti-SUMO-2/3 (Mab4971; Cell Signaling): rabbit polyclonal anti-ATRX (H-300; Santa Cruz Biotechnology), anti-Daxx (M-112; Santa Cruz Biotechnology), anti-NF200 (Pierce), anti-SUMO-1 (4930; Cell Signaling), anti-SUMO-2/3 (ab3742; Abcam), anti-ICP0 (Rab190), anti-VP16 (ab4808; Abcam), and anti-pan-HSV-1 (LSBio) were used. All secondary antibodies were Alexa Fluor-conjugated and were raised in goats (Invitrogen).

### Microscopy, imaging, and quantification

Observations and most image collections were performed using an inverted Cell Observer microscope (Zeiss) with a Plan-Apochromat ×100 N.A. 1.4 objective and a CoolSnap HQ2 camera from Molecular Dynamics (Ropper Scientific), or a Zeiss LSM 510 confocal microscope. Raw images were processed using the ImageJ software (NIH).

### PCR, RT-PCR, and RT-qPCR

Detection of *LacZ* transcripts in *in*1374-infected cultured neurons was performed using the FastLane Cell cDNA kit (Qiagen) using the following primers: *LacZ* fwd: 5’ GCAGCAACGAGACGTCA 3’, *LacZ* rev: 5’ GAAAGCTGGCTACAGGAAG 3’. Detection of HSV-1 genomes in cell and TG extracts was performed using primers targeting: TK fwd: 5’ GGAGGACAGACACATCGACC 3’, rev: 5’ CGAAAGCTGTCCCCAATCCT 3’ and LAT fwd: 5’ CCCACGTACTCCAAGAAGGC 3’, rev: 5’ AGACCCAAGCATAGAGAGCCAG 3’. RT-PCR for the detection of viral lytic mRNAs or LAT in human or mouse TG and cell extracts was performed using primers targeting: ICP0 fwd: 5’ GGT-GTA-CCT-GAT-AGT-GGG-CG 3’, rev: 5’ GCT-GAT-TGC-CCG-TCC-AGA-TA 3’; ICP4 fwd: 5’CGT-GGT-GGT-GCT-GTA-CTC-G 3’, rev: 5’ GCT-CGG-CGG-ACC-ACT-C 3’; ICP27 fwd: 5’ ATG-TGC-ATC-CAC-CAC-AAC-CT 3’, rev: 5’ TCC-TTA-ATG-TCC-GCC-AGA-CG 3’; UL30 fwd: 5’ TGT-TTC-GCG-TGT-GGG-ACA-TA 3’, rev: 5’ TTG-TCC-TTC-AGG-ACG-GCT-TC 3’; VP16 fwd: 5’ TGC-GGG-AGC-TAA-ACC-ACA-TT 3’, rev: 5’ TCC-AAC-TTC-GCC-CGA-ATC-AA 3’; and LAT (see above).

## Supporting Information

S1 Fig vDCP-NBs fusion in BJ human primary fibroblasts. *In vitro* model consisting of the infection of human primary fibroblasts with the replication-defective HSV-1 mutant *in*1374. This virus does not replicate at the temperature of 38.5°C and forms vDCP-NBs (Fig 1Ci) and [65]. (A) Immuno-FISH detection of vDCP-NBs at 2 dpi. (B) Representative images of small-sized vDCP-NBs observed between 6 and 24 hpi, and large-sized vDCP-NBs observed from 4 dpi onwards. Scale bar represents 5 μm. (C) Quantification of maximum, minimum, and average areas of vDCP-NBs per nucleus at different times pi (3 independent experiments). Cells were harvested at 6 h to 7 dpi and processed for FISH to visualize the viral genomes and measure the area of the spots. Bars represent the median, crosses represent the means. * p < = 0.05, ** p < = 0.01 (Student’s *t*-test). (D) Quantification of maximum, minimum, and average number of vDCP-NBs per nucleus at different times pi (3 independent experiments). Cells were harvested at 6 h to 7 dpi and processed for immuno-FISH to visualize the vDCP-NBs. Bars represent the median, crosses represent the means. ** p < = 0.01 (Student’s *t*-test). (E) Table showing the quantity of cells per sample during the whole experiment described in (F) from 6 hpi to 7 dpi. Quantification of the actin gene was used to determine the number of cells (three independent experiments).(F) Quantification by qPCR of the number of HSV-1 genomes in the cell population at different times pi (three independent experiments). Two genes, thymidine kinase (TK) and LAT were detected. No significant loss of viral genomes could be measured over the entire experiment (Student’s *t*-test).
(TIF)

S2 Fig Detection of viral proteins in ML-containing neurons at 28 dpi. Immuno-DNA-FISH showing HSV-1 genomes (red, 28 dpi), ICP4 (i) or ICP27 (ii) viral proteins (green), and cellular chromatin (DAPI, blue/grey). Dotted lines delimitate the nucleus. Scale bars represent 10 μm.
(TIF)

S3 Fig Daxx and ATRX signals increase in HSV-1 infected TG during acute infection. IF-FISH showing the Daxx (A) and ATRX (B) signals (green/grey) in non-infected (up) and HSV-1 (red) infected (down) cells. Nuclei are shown by DAPI staining (blue). Black and white images are also shown to facilitate Daxx and ATRX signals visualization. Arrows indicate infected neurons positive for HSV-1 genomes. Scale bar represents 10 μm.
(TIF)

S4 Fig Culture and characterization of primary mouse TG neurons. (A) Schematic protocol for the purification of TG neurons, and phase contrast images (lens 10 x) of cultures at different times post-preparation (i to iv). Phase contrast (v) and IF detection of neurons using neurofilaments antibody NF160 (vi) (lens 40 x). (B) IF detection of PML-NB-associated proteins, Daxx (i), ATRX (ii), SUMO-1 (iii), Daxx and PML (iv) in cultured neurons at d7. Neurofilaments are detected using the NF160 antibody (i, ii, and iii). DAPI is shown in blue. Scale bars represent 10 μm. (C) IF detection of viral proteins in cultured neurons infected by HSV-1wt for 24 h: ICP0 (i), ICP4 (ii), VP16 (iii), ICP27 (iv). DAPI is shown in blue/grey.
(TIF)

S5 Fig Infection of cultured mouse TG neurons with ICP0 or thymidine kinase (TK) mutant HSV-1. (A) Infection with ICP0 mutant dl1403. (i–vi) Immuno-FISH for the detection of PML (green or blue), Daxx, ATRX or SUMO-1 (green) and the HSV-1 genome (red). DAPI is shown in blue in (i), (ii) and (iii). (B) Infection with TK mutant 17/tBTK^-^. (i–iv) Immuno-FISH for the detection of PML (green or blue), Daxx, ATRX or SUMO-1 (green) and the HSV-1 genome (red). DAPI is shown in blue in (i). Scale bars represent 10 μm.
(TIF)

S6 Fig RC and vDCP-NBs are detected in primary mouse TG neurons infected with the *ts*K virus at the non permissive temperature. Neurons were infected with *ts*K at 38.5°C for 48 h. Immuno-FISH for the detection of PML (A and Bi), Daxx (Bii), ATRX (Biii) or SUMO-1 (Biv) (green) and the HSV-1 genome (red). (A) Detection of the RC pattern in small and large neurons. (B) Detection of vDCP-NBs in large neurons. Insets at the lower right corner of the images show the nuclei. Scale bars represent 10 μm.
(TIF)

S7 Fig Infection of mice with neuron replication-deficient thymidine kinase mutant virus. (A) Mice were infected with 10^6^ pfu of wt or TKDM21 (TK-) virus. Mice were sacrificed at various dpi then the lip area of virus inoculation was dissected in order to perform titrations. Three mice for each time point were analyzed. Results show means (± SD). (B) Mice were infected with 10^6^ pfu of wt or TKDM21 (TK-) virus. Mice were sacrificed at various dpi then TGs were harvested to perform titration assays. Three mice for each time point were analyzed. Results show means (± SD). (C) Quantification of viral genome patterns in neurons infected with the TKDM21 virus. Mice were infected with 10^6^ pfu of TKDM21 virus. Six dpi mice were sacrificed and immuno-FISH were performed on TG samples to detect HSV-1 genome patterns (RC or vDCP-NBs) and PML. Total TGs of 4 mice were thoroughly analyzed and only few neurons (48 in total) were detected with a positive signal for the viral genome.
(TIF)

S8 Fig Detection of Daxx and ATRX in RC-containing neurons following TSA treatment. (A) IF for detection of PML (green) and Daxx (i) or ATRX (ii) (red) in uninfected neurons treated with TSA [2μM] for 24 hours. Nuclei are detected with DAPI (blue). (B) and (C) RNA-DNA FISH combined with IF for detection of *LacZ* transcripts (blue), HSV-1 genomes (red), and Daxx (B), or ATRX (C) (green). Neurons were infected with *in*1374 at 38.5°C for 3 days then TSA [2μM] was added in the medium for 24 hours at 32°C. Different protein behaviors are shown. Scale bars represent 10 μm.
(TIF)
