# Supplementary figures and images for: Latency Entry of Herpes Simplex Virus 1 Is Determined by the Interaction of Its Genome with the Nuclear Environment
Source: PLoS Pathog. 2016 Sep 12;12(9):e1005834. doi: 10.1371/journal.ppat.1005834 (PMC5019400; doi:10.1371/journal.ppat.1005834)

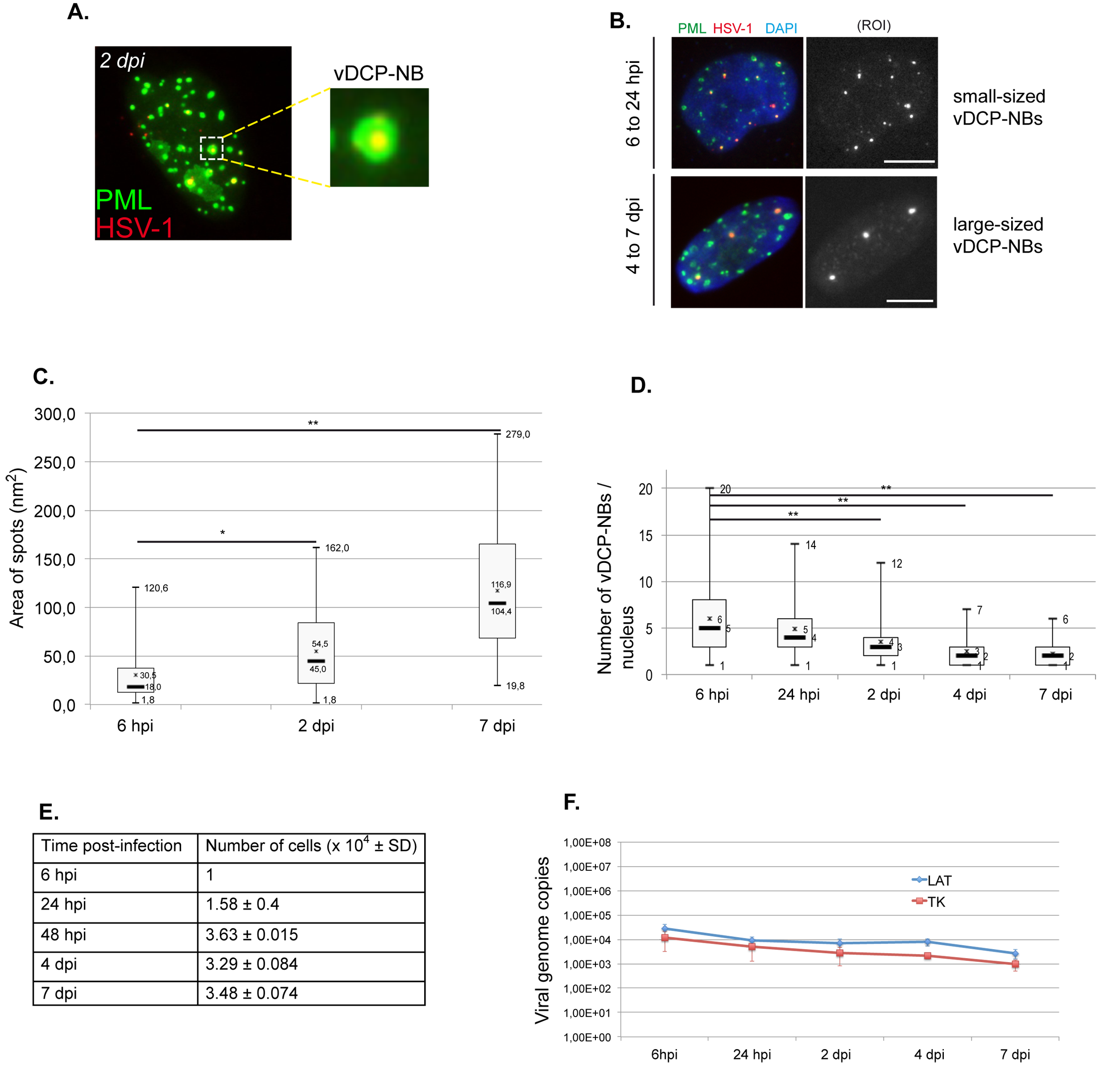

Supplement: S1 Fig — In vitro model consisting of the infection of human primary fibroblasts with the replication-defective HSV-1 mutant in1374. This virus does not replicate at the temperature of 38.5°C and forms vDCP-NBs (Fig 1Ci) and [65]. (A) Immuno-FISH detection of vDCP-NBs at 2 dpi. (B) Representative images of small-sized vDCP-NBs observed between 6 and 24 hpi, and large-sized vDCP-NBs observed from 4 dpi onwards. Scale bar represents 5 μm. (C) Quantification of maximum, minimum, and average areas of vDCP-NBs per nucleus at different times pi (3 independent experiments). Cells were harvested at 6 h to 7 dpi and processed for FISH to visualize the viral genomes and measure the area of the spots. Bars represent the median, crosses represent the means. * p < = 0.05, ** p < = 0.01 (Student’s t-test). (D) Quantification of maximum, minimum, and average number of vDCP-NBs per nucleus at different times pi (3 independent experiments). Cells were harvested at 6 h to 7 dpi and processed for immuno-FISH to visualize the vDCP-NBs. Bars represent the median, crosses represent the means. ** p < = 0.01 (Student’s t-test). (E) Table showing the quantity of cells per sample during the whole experiment described in (F) from 6 hpi to 7 dpi. Quantification of the actin gene was used to determine the number of cells (three independent experiments).(F) Quantification by qPCR of the number of HSV-1 genomes in the cell population at different times pi (three independent experiments). Two genes, thymidine kinase (TK) and LAT were detected. No significant loss of viral genomes could be measured over the entire experiment (Student’s t-test). (TIF) [file ppat.1005834.s001.tif]

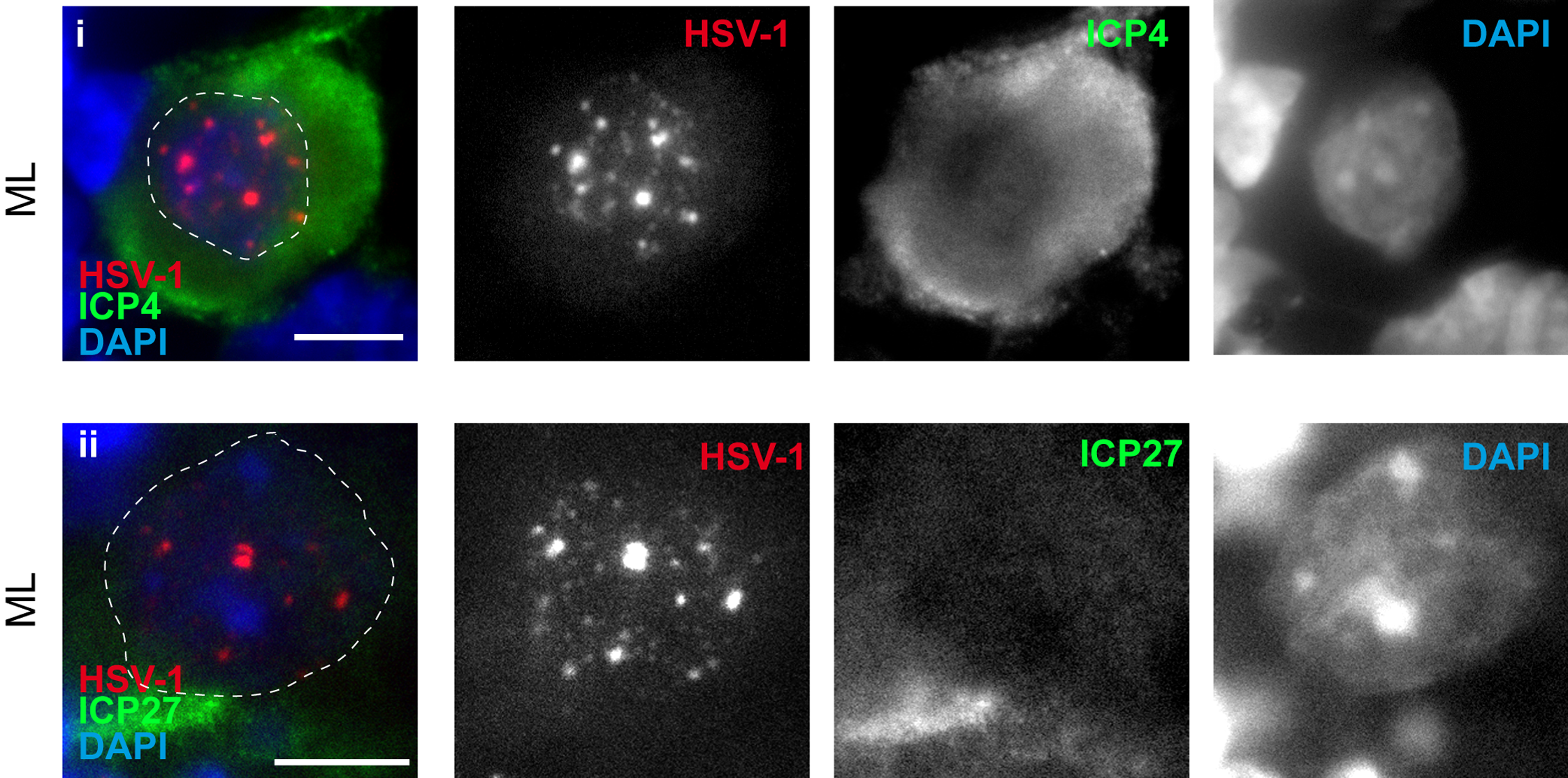

Supplement: S2 Fig — Immuno-DNA-FISH showing HSV-1 genomes (red, 28 dpi), ICP4 (i) or ICP27 (ii) viral proteins (green), and cellular chromatin (DAPI, blue/grey). Dotted lines delimitate the nucleus. Scale bars represent 10 μm. (TIF) [file ppat.1005834.s002.tif]

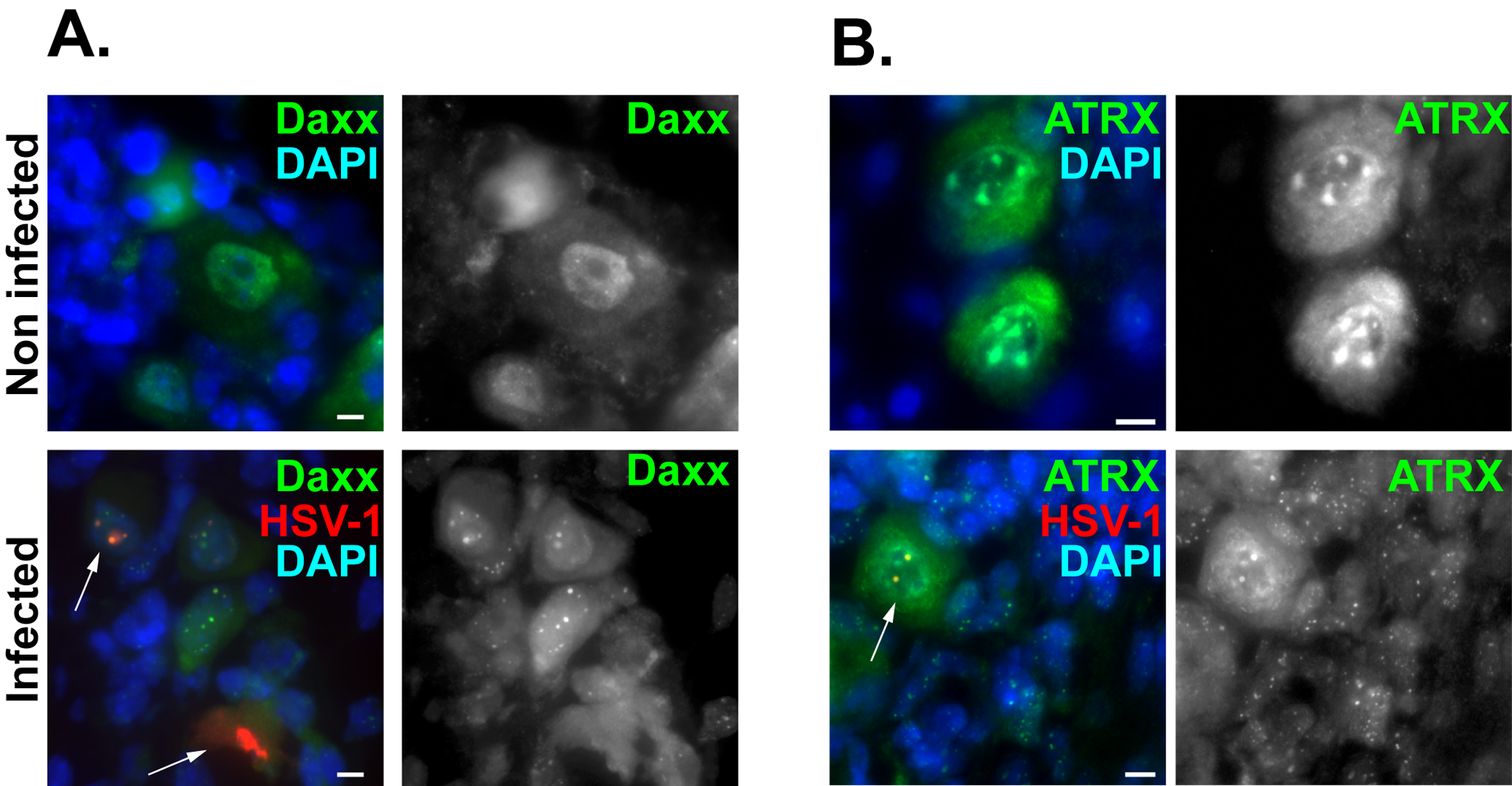

Supplement: S3 Fig — IF-FISH showing the Daxx (A) and ATRX (B) signals (green/grey) in non-infected (up) and HSV-1 (red) infected (down) cells. Nuclei are shown by DAPI staining (blue). Black and white images are also shown to facilitate Daxx and ATRX signals visualization. Arrows indicate infected neurons positive for HSV-1 genomes. Scale bar represents 10 μm. (TIF) [file ppat.1005834.s003.tif]

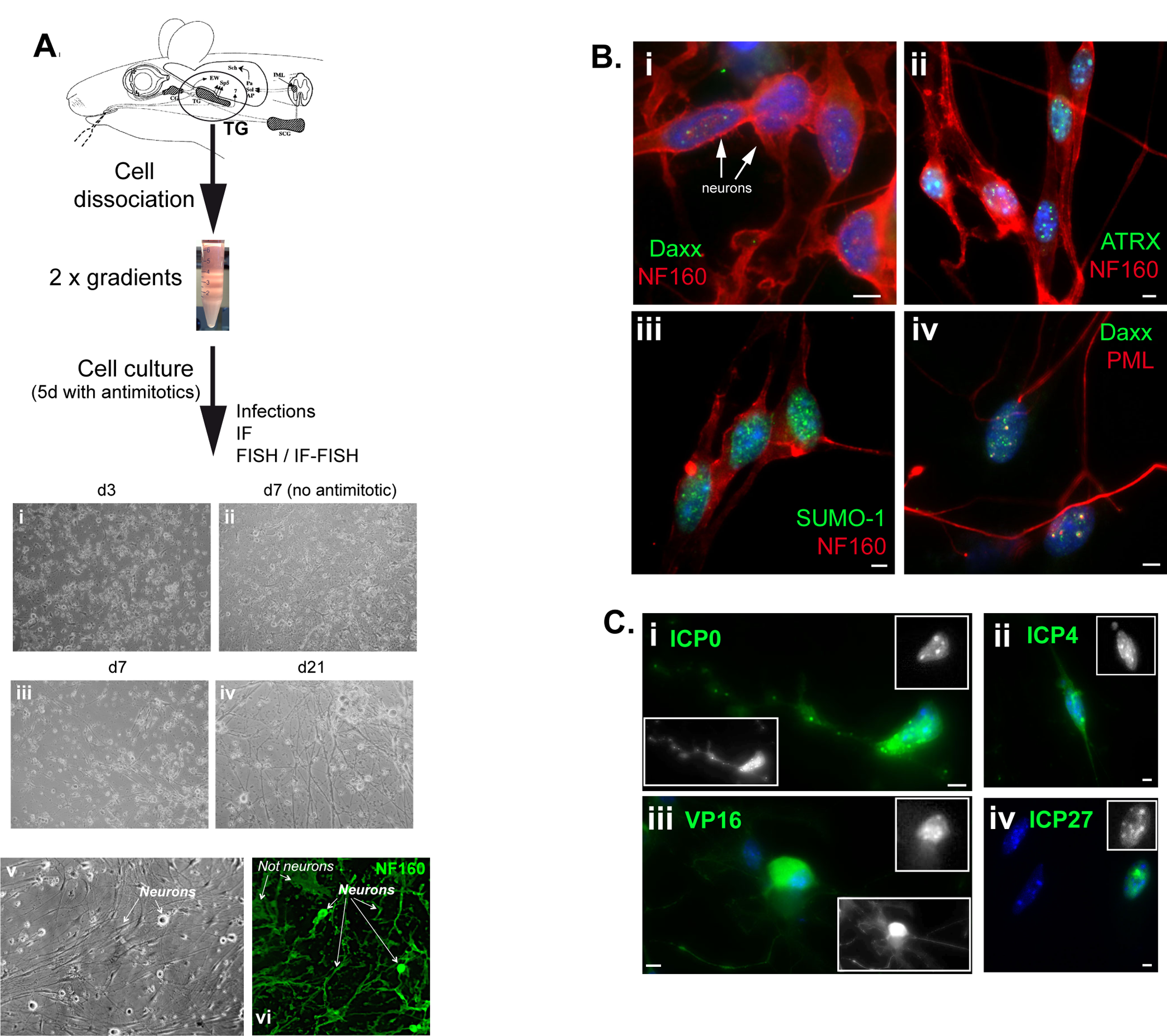

Supplement: S4 Fig — (A) Schematic protocol for the purification of TG neurons, and phase contrast images (lens 10 x) of cultures at different times post-preparation (i to iv). Phase contrast (v) and IF detection of neurons using neurofilaments antibody NF160 (vi) (lens 40 x). (B) IF detection of PML-NB-associated proteins, Daxx (i), ATRX (ii), SUMO-1 (iii), Daxx and PML (iv) in cultured neurons at d7. Neurofilaments are detected using the NF160 antibody (i, ii, and iii). DAPI is shown in blue. Scale bars represent 10 μm. (C) IF detection of viral proteins in cultured neurons infected by HSV-1wt for 24 h: ICP0 (i), ICP4 (ii), VP16 (iii), ICP27 (iv). DAPI is shown in blue/grey. (TIF) [file ppat.1005834.s004.tif]

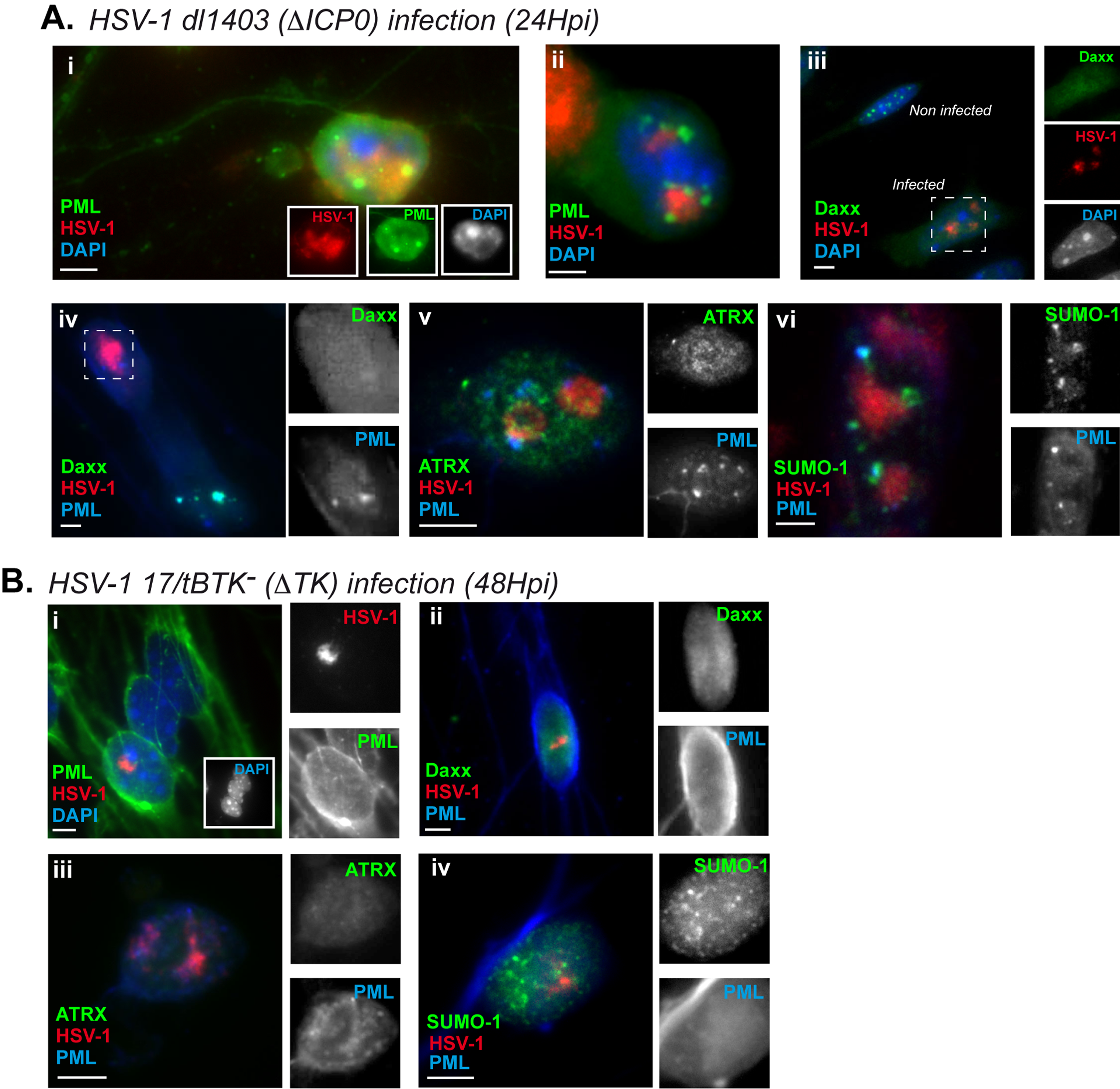

Supplement: S5 Fig — (A) Infection with ICP0 mutant dl1403. (i–vi) Immuno-FISH for the detection of PML (green or blue), Daxx, ATRX or SUMO-1 (green) and the HSV-1 genome (red). DAPI is shown in blue in (i), (ii) and (iii). (B) Infection with TK mutant 17/tBTK-. (i–iv) Immuno-FISH for the detection of PML (green or blue), Daxx, ATRX or SUMO-1 (green) and the HSV-1 genome (red). DAPI is shown in blue in (i). Scale bars represent 10 μm. (TIF) [file ppat.1005834.s005.tif]

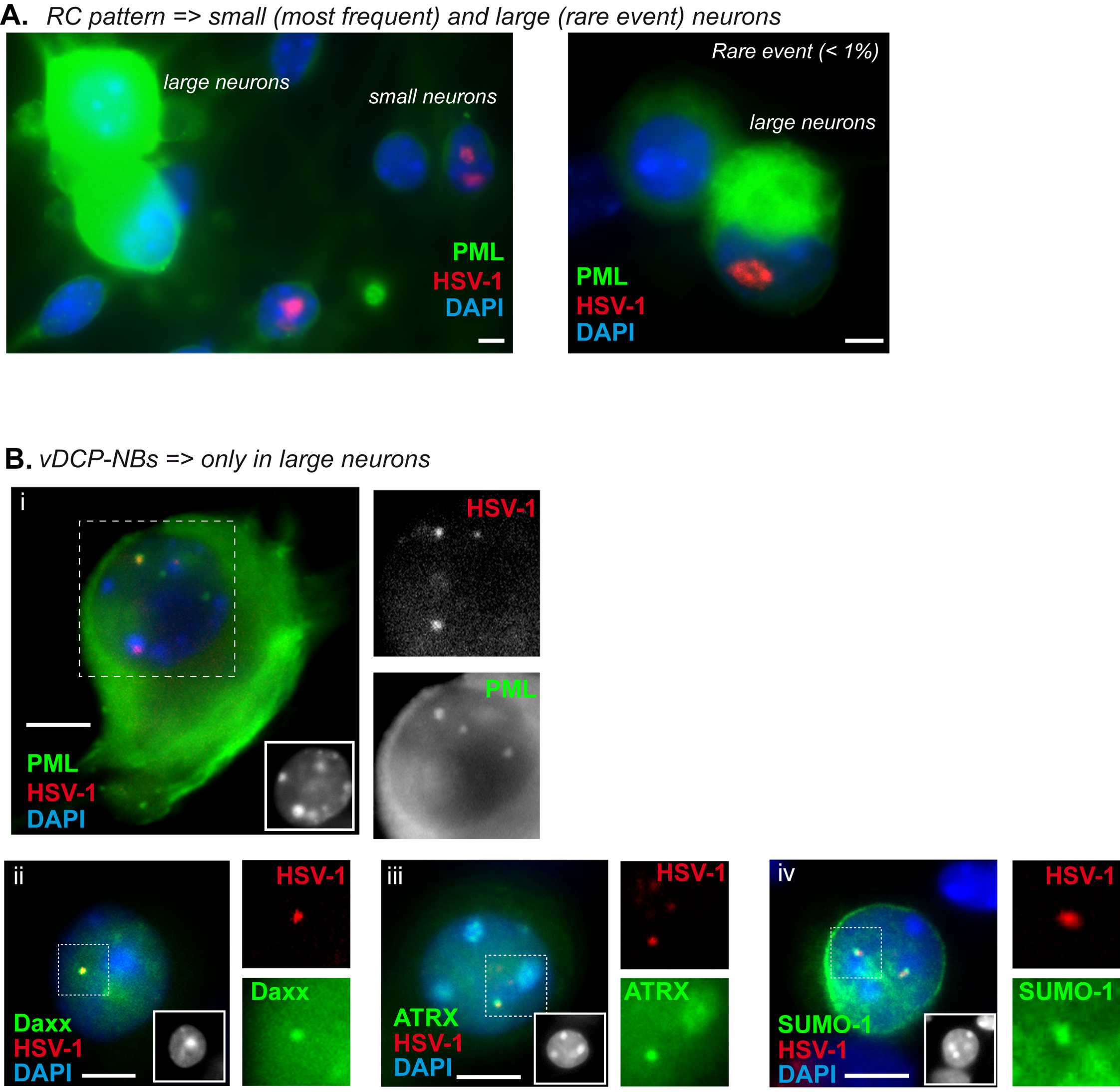

Supplement: S6 Fig — Neurons were infected with tsK at 38.5°C for 48 h. Immuno-FISH for the detection of PML (A and Bi), Daxx (Bii), ATRX (Biii) or SUMO-1 (Biv) (green) and the HSV-1 genome (red). (A) Detection of the RC pattern in small and large neurons. (B) Detection of vDCP-NBs in large neurons. Insets at the lower right corner of the images show the nuclei. Scale bars represent 10 μm. (TIF) [file ppat.1005834.s006.tif]

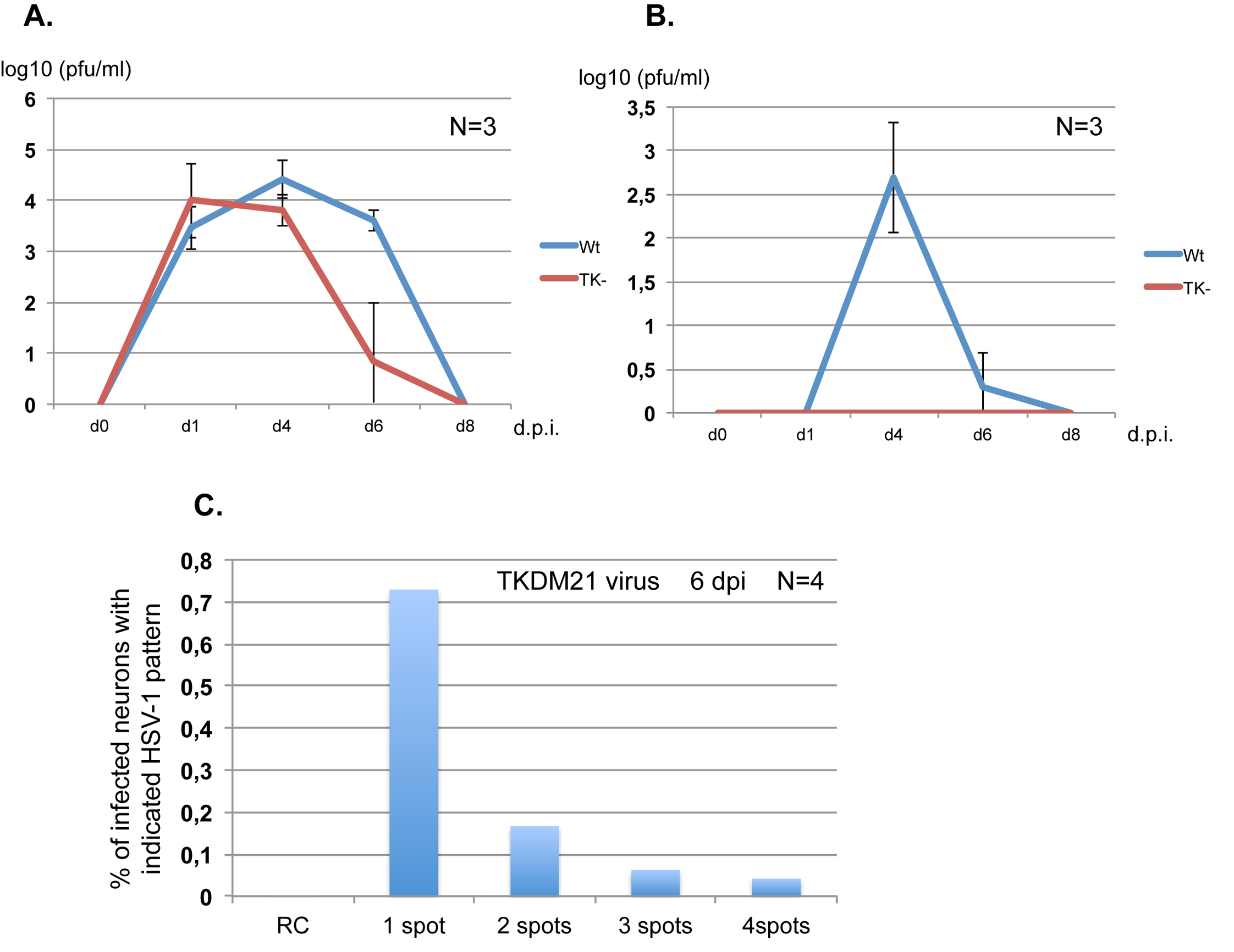

Supplement: S7 Fig — (A) Mice were infected with 106 pfu of wt or TKDM21 (TK-) virus. Mice were sacrificed at various dpi then the lip area of virus inoculation was dissected in order to perform titrations. Three mice for each time point were analyzed. Results show means (± SD). (B) Mice were infected with 106 pfu of wt or TKDM21 (TK-) virus. Mice were sacrificed at various dpi then TGs were harvested to perform titration assays. Three mice for each time point were analyzed. Results show means (± SD). (C) Quantification of viral genome patterns in neurons infected with the TKDM21 virus. Mice were infected with 106 pfu of TKDM21 virus. Six dpi mice were sacrificed and immuno-FISH were performed on TG samples to detect HSV-1 genome patterns (RC or vDCP-NBs) and PML. Total TGs of 4 mice were thoroughly analyzed and only few neurons (48 in total) were detected with a positive signal for the viral genome. (TIF) [file ppat.1005834.s007.tif]

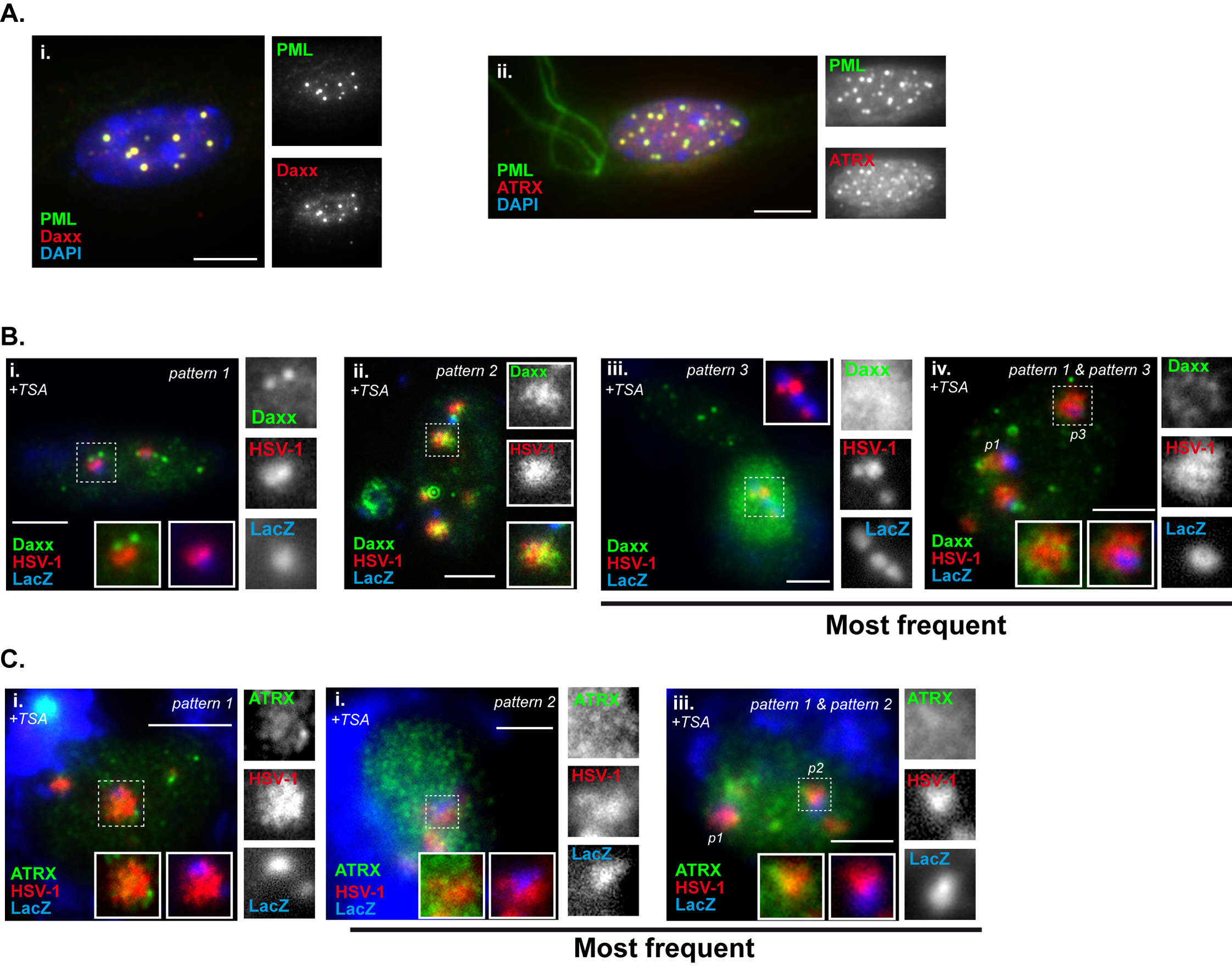

Supplement: S8 Fig — (A) IF for detection of PML (green) and Daxx (i) or ATRX (ii) (red) in uninfected neurons treated with TSA [2μM] for 24 hours. Nuclei are detected with DAPI (blue). (B) and (C) RNA-DNA FISH combined with IF for detection of LacZ transcripts (blue), HSV-1 genomes (red), and Daxx (B), or ATRX (C) (green). Neurons were infected with in1374 at 38.5°C for 3 days then TSA [2μM] was added in the medium for 24 hours at 32°C. Different protein behaviors are shown. Scale bars represent 10 μm. (TIF) [file ppat.1005834.s008.tif]
